# Magnetically “Programming” Cobalt‐Doped Iron Oxide Nanoparticles for Localized Induction Heating: Triggering a Collective Effect of Magnetic Moment Alignment on Demand

**DOI:** 10.1002/adma.202507158

**Published:** 2025-07-26

**Authors:** Theodor Raczka, Leoni Luthardt, Stephan Müssig, Noah Kent, Qianqian Lan, Thibaud Denneulin, Rafal E. Dunin‐Borkowski, Karl Mandel

**Affiliations:** ^1^ Chair ‘Particle‐based Materials Chemistry’ Section Materials Chemistry Department of Chemistry and Pharmacy Friedrich‐Alexander‐Universität Erlangen‐Nürnberg (FAU) Egerlandstraße 1 91058 Erlangen Germany; ^2^ Research Laboratory of Electronics Massachusetts Institute of Technology Cambridge MA 02139 USA; ^3^ McGovern Institute for Brain Research Massachusetts Institute of Technology Cambridge MA 02139 USA; ^4^ Ernst Ruska‐Centre for Microscopy and Spectroscopy with Electrons Forschungszentrum Jülich GmbH Wilhelm‐Johnen‐Straße 52425 Jülich Germany

**Keywords:** debonding on demand, induction heating, iron oxide nanoparticles, magnetic moment alignment, magnetic programming

## Abstract

Induction heating, a contactless and efficient method for generating heat via alternating magnetic fields (AMFs), has evolved from simple thermal applications to precise process control in fields like catalysis, self‐healing, and debonding. Magnetic nanoparticles (NPs) play a key role as heat mediators, with heating properties adjustable via composition, size, and interactions. However, spatially precise heat control remains challenging. Current strategies rely on external AMF adjustments or material modifications, but lack an inherent mechanism to predefine which particles or regions will be activated for induction heating, limiting applicability in structured materials or complex environments. Here, it is shown that pre‐magnetizing cobalt‐doped iron oxide NPs with a static magnetic field irreversibly enhances their heating rates by up to a factor of 40. This process permanently alters their magnetic properties, enabling selective heating independent of AMF modulation. The extent of activation scales with cobalt content, introducing a material‐intrinsic thermal switch. Furthermore, assembling these NPs into supraparticles facilitates integration into functional materials. By enabling spatially resolved and selective heat generation, this strategy advances the control of induction heating at the material level. It opens new possibilities for on‐demand, pre‐programmable, spatially resolved thermal activation in composite materials, smart adhesives, and targeted energy delivery in complex systems.

## Graphical abstract

This study demonstrates enhanced induction heating in cobalt‐doped iron oxide nanoparticles via specific pre‐magnetization, induced by the pre‐alignment of magnetic moments. By correlating Co content with magnetic properties, the research highlights how magnetic pre‐programming enables localized, on‐demand debonding in composites. These findings offer future applications in industrial recycling, emphasizing the potential of a switchable magnetic memory in advanced systems.

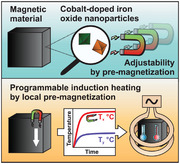

## 1 Introduction

Induction heating, that is the application of an alternating magnetic field (AMF) to a magnetic sample that is stimulated to generate heat,^[^ ^1^ ^]^ has manifested itself as a fast and contactless method for heating in various applications, although energy efficiency in colloidal systems may be reduced by impedance mismatch and other losses.^[^ ^2^ ^]^ However, recently, the trend of using induction heating is advancing to applications that go beyond the mere heating up of samples, but rather focus on the active control of processes, for example, debonding on demand,^[^ ^3^ ^]^ catalysis,^[^ ^4^, ^5^ ^]^ self‐healing and curing,^[^ ^6^ ^]^ or the activation of chemical reactions.^[^ ^7^ ^]^ Especially the use of nanoparticles (NPs) as effective heat mediators in induction heating has become very attractive,^[^ ^8^, ^9^ ^]^ in particular because of their exceptional spatial resolution that enables usage in locally precise scenarios.^[^ ^5^, ^10^ ^]^ Moreover, due to their small size, they can easily be integrated into various host matrices, thus allowing the transition of their particle‐based induction heating properties to use cases on an application‐oriented scale.^[^ ^11^, ^12^ ^]^ By the targeted manipulation of the size,^[^ ^13^, ^14^, ^15^ ^]^ morphology,^[^ ^9^, ^16^ ^]^ or composition of NPs,^[^ ^9^, ^15^, ^16^ ^]^ as well as their interactions with other species,^[^ ^17^ ^]^ it is possible to tune their response to an applied AMF in terms of their maximum induction heating temperature or exerted heating rate. At the same time, the induction heating performance of NPs can also be customized externally by specifically adjusting the AMF in frequency,^[^ ^18^ ^]^ amplitude,^[^ ^15^, ^19^ ^]^ or duration.^[^ ^20^ ^]^ However, in some cases, it is desirable to heat only specific NPs or localized regions. For example, in microfluidic systems, localized heating of NPs could enable precise control over biochemical reactions or phase transitions in targeted regions.^[^ ^21^ ^]^ Similarly, in smart adhesive applications, the ability to selectively activate induction heating in NPs for programmed debonding could allow for controlled disassembly or repair.^[^ ^22^ ^]^ The ability to selectively stimulate heating in certain NPs, particularly in a controlled, *ex post* manner, would provide an additional level of customization, allowing for adaptable and precise thermal control as needed in a given situation. This capability enhances the versatility of the system, enabling on‐demand heating in complex environments.

Some approaches to activate induction heating by employing remote stimuli or influences have already been reported,^[^ ^23^ ^]^ including environmental temperature,^[^ ^24^ ^]^ pH manipulation,^[^ ^25^ ^]^ mechanical triggers,^[^ ^25^ ^]^ and combinations of magnetic samples with light‐triggered species.^[^ ^26^ ^]^ However, all these stimuli come with certain drawbacks, as chemical manipulation as well as thermal and mechanical influences may change the overall sample composition, while light‐triggered events are not feasible when the NPs are integrated inside a matrix. To address these limitations, we propose using a static magnetic field generated by an external magnet as a trigger. The advantage of this approach is that the external trigger can be applied remotely and locally, even when the NPs are already integrated into a matrix. This allows the application of the magnetic field at any stage of the NP processing or implementation. Additionally, NPs with high coercivity can be made more responsive to the AMF by magnetizing them, thus allowing induction heating to higher temperatures and enhancing their utility in various applications.^[^ ^11^, ^15^ ^]^

**Figure 1:**
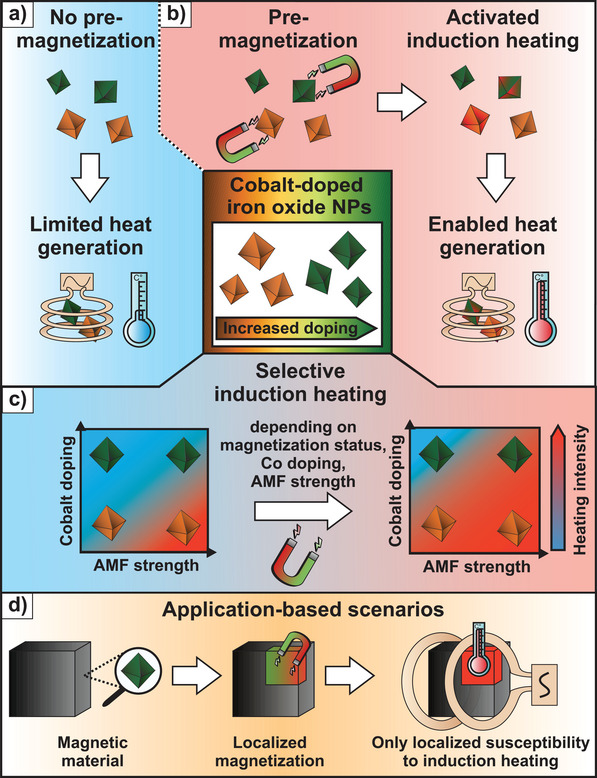
Schematic representation of the working principle of magnetically programmable Co‐doped iron oxide NPs. a) When the NPs are not magnetized, they produce limited heat upon exposure to an AMF from the induction heating device. b) When pre‐magnetized by an external static magnet, the NPs heat more efficiently upon exposure to an AMF. c) The response of the NPs to a given AMF amplitude is highly dependent on the level of Co doping and AMF strength, allowing selective induction heating with various NP types. In this way, pre‐magnetization and low AMF exposure excite induction heating only in lower Co‐doped NPs, while higher Co‐doped iron oxide NPs require higher AMF amplitudes. d) This effect can be made of use in application‐based scenarios, where SPs integrated into a matrix material are locally magnetized and thus produce heat in a spatially resolved manner when exposed to an AMF.

In this work, the induction heating characteristics of iron oxide NPs doped with different amounts of cobalt (Co) are enhanced manifold by applying external magnetic fields before the induction heating procedure. With this proof‐of‐concept, knowledge about magnetic field modulation in terms of field strength can be exploited for spatially resolved or selective heating. Without prior exposure to a static magnetic field, the heating efficiency and response to an AMF of such NPs are deliberately low (**Figure** **1**a). However, when the NPs are first subjected to a static magnetic field and subsequently to an AMF, their induction heating rates can be elevated up to a factor of 40, while the maximum heating temperature is increased up to 10‐fold (Figure 1b). Hence, their magnetic response can be controlled in two steps: on the one hand, internally by the performed NP synthesis and the amount of doping, and on the other hand, externally by applying a static magnetic field. Therefore, targeted external pre‐magnetization is utilized as an active strategy to irreversibly enhance induction heating with high spatial resolution in previously poorly excitable magnetic materials. The selective induction heating of differently doped NPs can be made use of, as NPs with a lower Co doping display enhanced induction heating rates at lower AMFs after pre‐magnetization compared to higher doped NPs (Figure 1c). Therefore, the benefit of selective heating depending on the magnetization status of the NPs and the applied AMF is underlined. Furthermore, microparticles, so‐called supraparticles (SPs), are synthesized from Co‐doped iron oxide NPs via spray‐drying, to allow their integration into matrices in applied contexts, where they are tested as actively triggerable switches in on‐demand debonding processes (Figure 1d).

## 2 Results and Discussion

### 2.1 Characterization of Co‐doped Iron Oxide Nanoparticles

**Figure 2:**
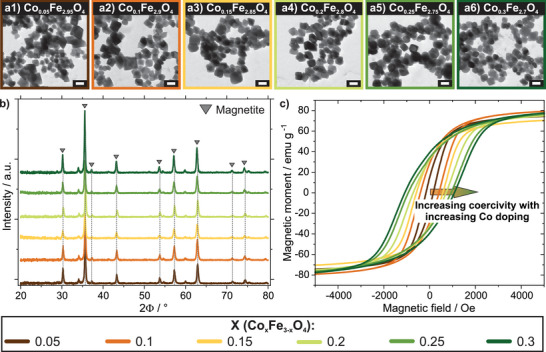
Structural and magnetic characterization of six different Co‐doped FIONs of the type Co_x_Fe_3‐x_O_4_, with X ranging from 0.05 to 0.3. a) TEM images of all six different Co dopings prepared for FIONs reveal a comparable size and morphology for all species. Scale bars represent 50 nm. b) XRD patterns for all NP types indicate a magnetite/maghemite structure for all NP types. c) VSM curves for all NP species demonstrating an increasing coercivity with an increasing amount of Co doping in FIONs. For clarity, initial curves are not depicted.

For magnetic programming of NPs, Co‐doped ferrimagnetic iron oxide NPs (FIONs) have been prepared by a modified oxidative precipitation method.^[^ ^11^, ^27^ ^]^ It is important to mention that no magnetic field was present during the synthesis procedure to avoid unwanted pre‐magnetization. In this manner, six differently Co‐doped NP species of the type Co_x_Fe_3‐x_O_4_ were synthesized, with X ranging from 0.05 to 0.3 in steps of 0.05. The dopant concentration is confirmed by inductively coupled plasma atomic emission spectroscopy (ICP‐AES) measurements performed on three exemplary samples (see Table S1, Supporting Information). Transmission electron microscopy (TEM) images reveal that all resulting NP types are roughly in the same size range with a mean diameter of ≈40 ± 12 nm (**Figure** **2**a, error describes the mean standard deviation, for size distribution plots see Figure S1, Supporting Information). Throughout all NP species, the resulting morphology is in a comparable octahedral shape as expected for this synthesis process.^[^ ^27^ ^]^ Increasing Co doping alters the NP morphology, leading to an increase in spherical character.^[^ ^28^, ^29^, ^30^ ^]^ SEM‐EDX data reveals uniform Co distribution, shown exemplarily for the lowest and highest doped samples on three individual spots, respectively (Figure S2, Supporting Information).

X‐ray diffraction (XRD) patterns recorded for all six species, respectively, consistently reveal a magnetite/maghemite structure (Figure 2b), indicated by characteristic reflexes (positions at 2Θ: 30.3°, 35.6°, 37.2°, 43.3°, 53.7°, 57.3°, 62.9°, 71.3°, 74.4°).^[^ ^31^ ^]^ It is notable that with increasing Co doping, the reflexes are shifted to lower values of 2Θ, as described in literature (see Supporting Information for a more detailed explanation).^[^ ^29^, ^30^, ^32^ ^]^ Therefore, the successful integration of Co into the magnetite structure is demonstrated.^[^ ^29^ ^]^

With an increasing amount of Co doping in FIONs, it is shown by vibrating sample magnetometer (VSM) measurements that the coercivity increases as well (Figure 2c). In this regard, while the Co_0.05_Fe_2.95_O_4_ NPs only display a coercivity of 160 Oe, the coercivity is enhanced almost seven‐fold to 1080 Oe for Co_0.3_Fe_2.7_O_4_. For all samples, the saturation magnetization stays roughly the same at a value around 80 emu g^−1^ (see Figure S3, Supporting Information for magnetization curves recorded from −30 to 30 kOe). The exact values for coercivity as well as saturation magnetization for every Co‐doped FION sample are displayed in Table S2 (Supporting Information). A comparison with literature data for similarly sized Co_X_Fe_3‐X_O_4_ NPs further confirms the high magnetic quality of the synthesized NPs (see Discussion of data displayed in Table S2, Supporting Information).

The phenomenon of increasing coercivity with an increasing amount of Co doping in NPs is known in the literature.^[^ ^29^, ^32^, ^33^ ^]^ Doping NPs with Co enhances their magnetic anisotropy, causing their magnetic moments to align strongly in a certain direction. Therefore, the energy needed to rotate these magnetic moments out of their alignment direction is increased.^[^ ^32^ ^]^ The magnetocrystalline anisotropy is magnified by increased Co doping, as spin‐orbit‐coupling is enhanced by the incorporation of Co due to three unpaired electrons.^[^ ^34^ ^]^ Hence, the effective anisotropy constant *K_eff_* is directly proportional to the observed coercivity, as per Equation 1:^[^ ^35^ ^]^

(1) $$K_{\mathrm{eff}}=\frac{H_{c}M_{s}}{0.96}$$

where *H_C´_* is the measured coercivity in Oe and *M_S_* is the saturation magnetization in emu g^−1^. Following the increased coercivity and anisotropy, *K_eff_* is magnified by a factor of approximately seven when the Co doping is increased from 0.05 to 0.3 (see Table S2, Supporting Information).

### 2.2 Induction Heating and Magnetic Behavior of Co‐Doped Iron Oxide Nanoparticles Before and After Magnetization

**Figure 3:**
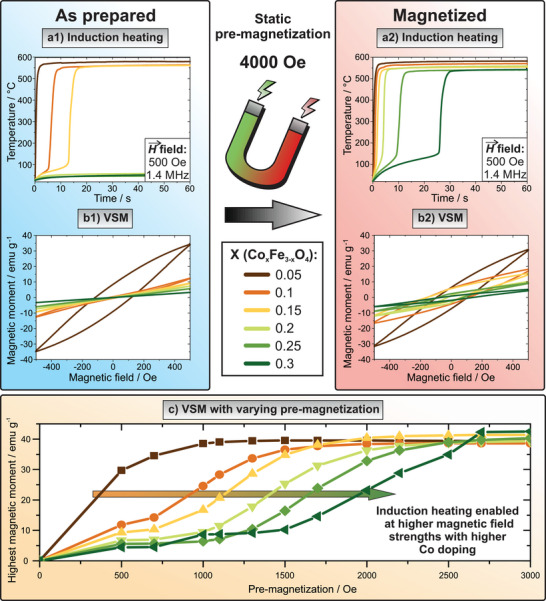
Magnetic characterization of Co‐doped FIONs before 1) and after 2) pre‐magnetization with a static magnet in terms of induction heating a) and static (DC) VSM b). For clarity, initial curves are not depicted. For all induction heating curves, a field amplitude of 500 Oe and an AMF frequency of 1419 kHz were employed. While before pre‐magnetization, induction heating to higher temperatures is only induced for lower Co dopings, all samples are triggered to produce heat after exposure to a static magnet. When the individual samples are pre‐magnetized to different values c), their maximum magnetic moment is enhanced with increasing magnetization until it reaches a plateau. Here, it becomes apparent that the higher the Co doping in FIONs is, the higher is the magnetic field that is required for pre‐magnetization to reach full saturation, resulting in enhanced induction heating rates.

When all Co‐doped FIONs species are exposed to an AMF with an amplitude of 500 Oe, only the three species with the lowest dopings (X ranging from 0.05 to 0.15) produce heat in their pristine state. Co_0.05_Fe_2.95_O_4_ NPs are heated to high temperatures instantly when exposed to an AMF, while those with X = 0.1 enhance their heating rate after 5 s and those with X = 0.15 only after 12 s (**Figure** **3**a‐1). For the three NP species with higher dopings, no significant induction heating is measurable. This behavior is in accordance with hysteresis curves recorded for a static field between −500 and 500 Oe to simulate the AMF in the induction heating device (Figure 3a‐2). Although such quasi‐static measurements do not fully replicate the dynamic conditions during induction heating, they provide a useful approximation of the NPs’ magnetic switching behavior and energy dissipation potential. It is known that dynamic losses under alternating magnetic fields may differ in magnitude, yet typically follow the same qualitative trends observed in static loops.^[^ ^11^, ^36^ ^]^ For X = 0.05, a fully developed hysteresis loop is recorded, with a coercivity of 120 Oe and a saturation magnetization of 34 emu g^−1^. For X = 0.1 and 0.15, coercivities of 20 and 15 Oe, respectively, are noted, with corresponding saturation magnetizations of 12 and 9 emu g^−1^. For NPs with higher dopings, no significant coercivity is measurable, while the saturation magnetization remains low. The reduced coercivity observed despite higher Co doping levels is due to incomplete magnetization of the NPs, which also hampers the induction heating at the given amplitude of 500 Oe. The diminished magnetizability for higher Co‐doped NPs is attributed to their increased anisotropy, which leads to reduced magnetic susceptibility and an enhanced energy requirement to reverse their magnetic orientation. This prevents the NPs from dynamically aligning with the applied AMF, thereby reducing their induction heating capability.^[^ ^13^ ^]^

However, the observed induction heating behavior of the Co‐doped NPs changes drastically once they are magnetized by a static magnet with a magnetic field strength of ≈4000 Oe. This irreversible magnetization results in enhanced induction heating performance at lower AMF strengths compared to the unmagnetized pristine state. It is important to mention that the improved induction heating is observed after pre‐magnetization rather than during application of the external magnetic field. In this manner, the magnetized state can be programmed into the NPs and preserved indefinitely after one‐time exposure to an external static magnetic field, making enhanced induction heating rates feasible at any later point.

Doing so, even NPs doped with a higher amount of Co, meaning X = 0.2 to 0.3, heat inductively, although no heating was observed in the pristine state. Now, NPs with X = 0.2 are programmed to heat after 3 s, X = 0.25 after 9 s, and even X = 0.3 after 25 s, while NPs doped with lower amounts of Co heat almost instantly (Figure 3a‐2). This performance is also valid when considering the induction heating curves for smaller field amplitudes, for example 250 Oe (see Figure S4, Supporting Information), and is further visualized by comparing the induction heating curves of all respective Co‐doped species before and after magnetization at different field amplitudes (see Figure S5, Supporting Information). This improved induction heating behavior is reflected in VSM data recorded after pre‐magnetization, now revealing increased coercivity and hysteresis area for all NP species (Figure 3b‐2, for detailed values of the coercivity and saturation magnetization measured before and after magnetization in the minor VSM curves see Table S3, Supporting Information). Except for the lowest Co doping (X = 0.05), pre‐magnetization enhances magnetizability by reducing the minimum field required for substantial heat production, improving overall induction heating performance. This is further supported when considering the direct comparison of VSM curves (−500–500 Oe) before and after magnetization with 4000 Oe, revealing an increased hysteresis area and corresponding induction heating enhancement (**Table** **1** and see Figure S6, Supporting Information for comparison of VSM curves and plots of the hysteresis area with discussion of the values for X = 0.05).^[^ ^13^, ^19^, ^37^ ^]^ Hence, prior magnetization of Co‐doped FIONs acts as a magnetic on‐switch for induction heating, especially when considering higher Co dopings. The energy input for every Co‐doped FION species at the maximum AMF amplitude was calculated in Oe s^−1^ K^−1^ and displayed in the Supporting Information (Table S4, Supporting Information). The benefit of pre‐magnetization becomes obvious when comparing the mean energy input of 1.26 Oe s^−1^K^−1^ for pre‐magnetized NPs with 183 Oe s^−1^K^−1^ for pristine NPs.

**Table 1:** Hysteresis areas for VSM curves recorded between −500 and 500 Oe for unmagnetized and pre‐magnetized states of Co‐doped FIONs of the type Co_x_Fe_3‐x_O_4_, given in A^2^ m kg^−1^.

X=	0.05	0.10	0.15	0.20	0.25	0.30
**Unmagnetized**	11 471	1058	553	226	110	36
**Pre‐magnetized**	8832	6798	4594	1728	1923	1667

The calculated hysteresis areas from the static loops (−500 to 500 Oe) clearly illustrate the changes in magnetic energy dissipation caused by pre‐magnetization. The pronounced increase in loop area for Co contents X ≥ 0.1 correlates well with the improved heating performance. As the hysteresis area reflects the energy lost per magnetic cycle, this parameter provides a direct link between magnetic and thermal behavior under alternating magnetic fields.

While these measurements were performed under static field conditions, they already allow qualitative predictions for the dynamic heating behavior. To further support this interpretation and account for frequency‐dependent effects, additional dynamic hysteresis loops were recorded at 1000 Hz (see Figure S7 and Table S5, Supporting Information). These loops confirm the trend observed in the DC curves: NPs with low Co doping exhibit significant loop opening, while those with higher doping remain nearly lossless unless pre‐magnetized. However, such qualitative comparisons between static and dynamic hysteresis behavior may not be valid for superparamagnetic or very small ferrimagnetic NPs with narrow loops, where heating mechanisms can differ significantly.^[^ ^38^ ^]^

We hypothesize that the observed programmable enhancement of induction heating in Co‐doped FIONs under varying AMF amplitudes arises primarily from differences in magnetic coercivity among the samples. Coercivity, which determines how easily magnetic moments can be reversed by an external field, decreases as the NPs heat up. This temperature‐dependent coercivity reduction is demonstrated in the Supporting Information for temperatures between 275 and 400 K (Figure S8, Supporting Information) and is consistent with findings in other inductively heatable NP systems.^[^ ^15^ ^]^ When the coercivity drops below the amplitude of the applied AMF (500 Oe), magnetic switching becomes energetically favorable, allowing the NPs to dissipate energy effectively and heat up. This heating continues until the particles approach their Curie temperature, which lies between 570 and 600 °C for all samples, as confirmed by thermogravimetric analysis (TGA) (Figure S9, Supporting Information). We observe that samples with higher Co doping exhibit slightly lower Curie temperatures, resulting in moderately reduced maximum heating temperatures for these samples. The initial coercivity, controlled by the level of Co doping, governs the onset of heating behavior. Samples with low initial coercivity are immediately responsive to the applied AMF, generating heat from the start. In contrast, samples with higher initial coercivity require an increase in temperature (and thus a reduction in coercivity) before significant heating begins. This explains the delayed heating rates observed in some samples. The exact values for the coercivity measured for all Co‐doped samples at different temperatures are displayed in the Table S6 (Supporting Information). It is further shown in comparison with undoped as well as Ni‐ and Mn‐doped species in the Supporting Information that the sufficiently large coercivity of Co‐doped FIONs is the crucial factor for the programmable switching upon pre‐magnetization (Figure S10, Supporting Information). In all other cases, induction heating is activated independently of magnetic programming.

This temperature‐dependent behavior is linked to overcoming the magnetocrystalline anisotropy barrier. The effective anisotropy constant decreases with increasing temperature for all samples (for exact values, see Table S7, Supporting Information), effectively lowering the energy barrier for magnetic moment reorientation. Literature confirms that the effective magnetocrystalline anisotropy diminishes as temperature rises, facilitating magnetic switching beyond a critical thermal threshold.^[^ ^39^, ^40^ ^]^

This mechanism offers a compelling explanation for the two‐step heating behavior observed for the X =  0.3 sample in Figure 3a‐2. Initially, the temperature increases only slowly, reflecting a regime in which few magnetic moments contribute to hysteretic switching. Once the system reaches ≈150 °C, a sharp increase in heating rate up to ≈500 °C is observed, likely due to cooperative switching of a larger number of moments. We interpret this behavior as resulting from the altered anisotropy landscape following pre‐magnetization, which enables more efficient energy dissipation under the AMF beyond this threshold. This two‐step behavior is reproducible across different samples and experimental conditions, as seen in Figures S4, S5, S14 (Supporting Information). Despite differences in Co doping and magnetic anisotropy, all samples exhibit a similar heating threshold around 150 °C. This temperature likely represents a critical thermal activation point at which magnetic relaxation accelerates and energy barriers associated with anisotropy are overcome, enabling more efficient magnetization switching and dynamic energy dissipation under AMF exposure.^[^ ^23^, ^40^, ^41^ ^]^

High‐temperature static VSM measurements at 150 °C as well as at 500 °C for the fully heated state (Figure S11, Supporting Information, exemplary for Co_x_Fe_3‐x_O_4_ with X = 0.05 and 0.3) confirm this interpretation: both saturation magnetization and coercivity decrease with temperature, facilitating magnetic moment alignment with the AMF and indicating successful overcoming of the effective thermal anisotropy barrier. Finally, it is worth noting that annealing the Co‐doped FIONs at their Curie temperature can alter the effective anisotropy barrier and effectively “turn off” the magnetically switchable induction heating properties. This phenomenon is discussed in detail in the Supporting Information (see also Table S8 and Figure S12, Supporting Information). Please also refer to the Supporting Information for evaluation of the durability of the heating behavior over multiple induction heating cycles (Figure S13, Supporting Information).

Additionally, it has been investigated which magnetic field is needed for the individual Co‐doped NPs to be programmed to achieve their maximum induction heating performance, or in other words, to be magnetized to saturation. For this purpose, hysteresis curves between −500 and 500 Oe (to simulate the AMF of the induction heating device) have been recorded after respective pre‐magnetization steps between 500 and 5000 Oe. Doing so, it has been evaluated which static field strength for pre‐magnetization is necessary so that the highest measured magnetic moment does not change anymore, and the plot of this value against the pre‐magnetization value reaches a plateau (Figure 3c). The resulting trend revealed that with higher amounts of Co doping, higher static magnetization fields are required to reach saturation. All species were magnetically saturated with a pre‐magnetization of 2700 Oe or less, which renders the chosen value of 4000 Oe here sufficient. This is following induction heating curves obtained from Co_0.3_Fe_2.7_O_4_ NPs after different pre‐magnetization values, where improved induction heating is prompted after a minimum prior magnetization with 2500 Oe (see Figure S14, Supporting Information). Co_0.05_Fe_2.95_O_4_ NPs reach their plateau at the lowest value of a static field with ≈1100 Oe. However, at a pre‐magnetization value of 500 Oe, they are already close to magnetic saturation, therefore already allowing induction heating in their pristine state. For the other species it is observed that the lower the Co doping is, the higher the magnetic moment that is reached at fixed respective pre‐magnetization values (e.g., in the presented setup, a magnetic moment higher than 25 emu g^−1^ is reached with a pre‐magnetization of 500 Oe for X = 0.05, 1100 Oe for X = 0.1, 1300 Oe for X = 0.15, 1500 Oe for X = 0.2, 2000 Oe for X = 0.25, and 2200 Oe for X = 0.3). The corresponding hysteresis curves measured between −500 and 500 Oe for every Co‐doped species are following these findings, showing that with lower Co doping, less pre‐magnetization strength is needed to achieve a hysteresis loop that remains unchanged with higher pre‐magnetization values (see Figure S15, Supporting Information, with additional discussion of the form of VSM curves).

### 2.3 Mechanism of the Magnetically Switchable Induction Heating Performance

**Figure 4:**
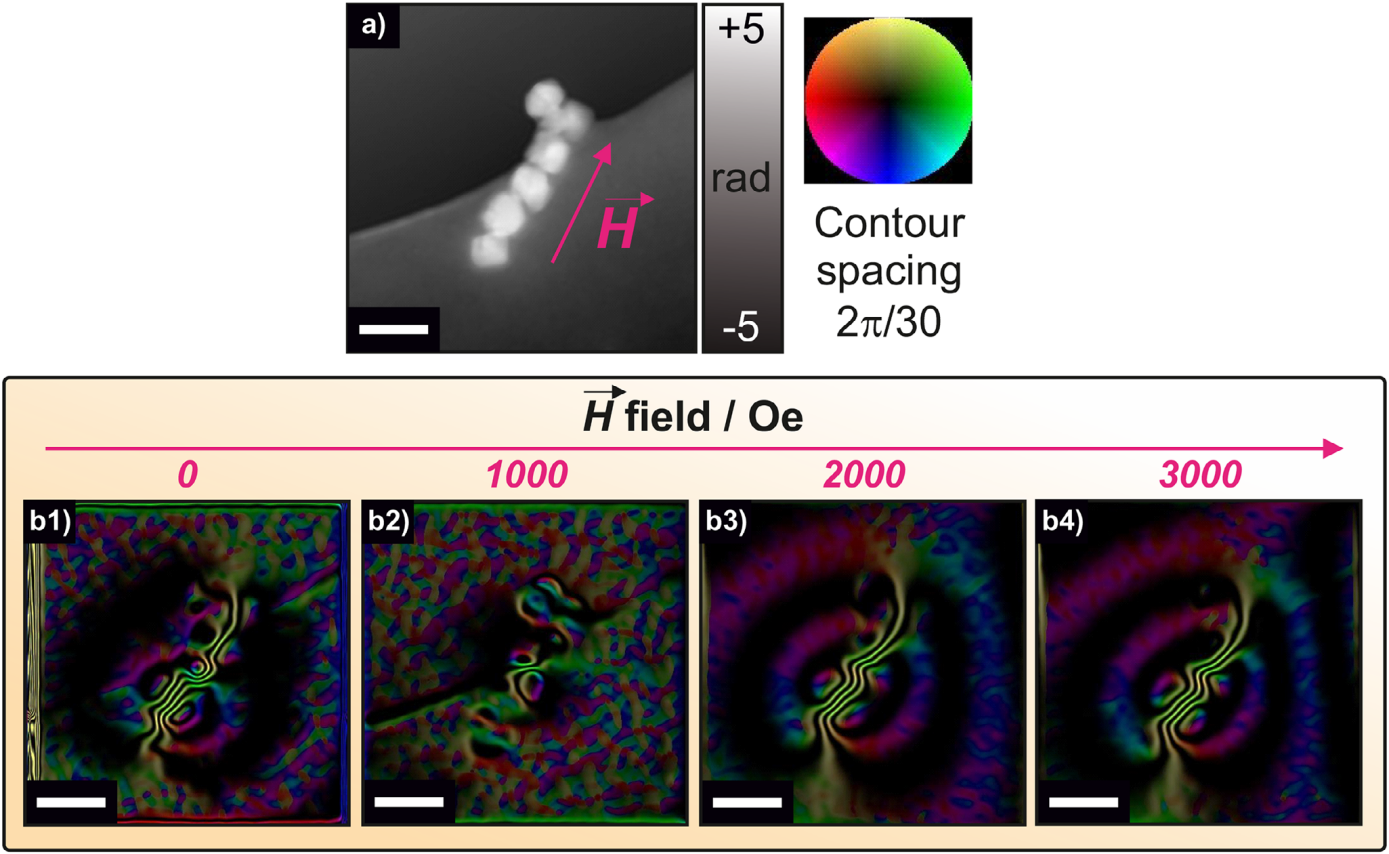
Projected in‐plane induction (B‐field) field distribution of Co_0.3_Fe_2.7_O_4_ NPs. a) The electrostatic phase image reveals the morphology and arrangement of Co_0.3_Fe_2.7_O_4_ NPs. b) Magnetic contour images extracted from magnetic phase images obtained via off‐axis electron holography, recorded in the remanent state after applying an external magnetizing field of 0 (1), 1000 (2), 2000 (3), and 3000 Oe (4), respectively. The applied field direction is along the NP chain axis (indicated by the red arrow in a)). The color wheel indicates the direction of the projected magnetic induction, while the density of the contour lines indicates the strength of the magnetic field. As the external field increases, the initially uniform magnetic moments of the Co‐doped FIONs progressively rearrange and tend to align along the direction of the applied field until approaching saturation. Scale bars correspond to 100 nm.

We hypothesize that the ability to modulate the induction heating behavior of Co‐doped FIONs is attributed to the pre‐alignment of magnetic domains inside the NPs once they are exposed to an external static magnetic field, as we exclude phenomena such as field‐induced chain formation due to the immobilized, powder‐like nature of our samples. Unlike colloidal systems or biological matrices, where dipolar interactions can lead to reversible self‐assembly into linear chains under AMF exposure, our dry and compacted particle ensembles do not permit such reorganization during heating experiments.^[^ ^42^ ^]^ To investigate this effect on the nanoscale, off‐axis electron holography (OAEH) was employed (**Figure** **4**). Here, NPs were exposed to external magnetic fields of 0, 1000, 2000, and 3000 Oe, which were then removed before imaging. It is important to emphasize that all images and maps represent the remanent magnetic state, meaning the internal magnetic configuration after the external field was switched off. The corresponding electrostatic phase, magnetic phase images, and magnetic induction maps were reconstructed from the holograms and analyzed accordingly.

The experiment was carried out using Co_0.3_Fe_2.7_O_4_ NPs arranged along the direction of the external magnetic field, as shown in the red arrow in Figure 4a (magnetic phase images see Figure S16, Supporting Information). With the absence of a magnetic field, the magnetic induction map reveals an intrinsic magnetic moment, likely resulting from a naturally preferred orientation of magnetic moments, albeit in a non‐uniform pre‐arranged state (Figure 4b‐1). With the increase of the magnetic field, the magnetic induction maps show that the magnetic moments within the NPs rearranged and progressively aligned along the external field direction (Figure 4b‐2). Although only discrete field values were applied, the data suggests that magnetic saturation is approached between 2000 and 3000 Oe (Figure 4b‐3–b4). This saturation correlates well with the minimum magnetic field required to enhance the induction heating performance of these NPs. Therefore, while an exact critical field for stable oriented structures cannot be precisely determined from these measurements due to the limited field increments, the observed magnetic response supports the macroscopic heating behavior. An analysis of the lowest doped sample, Co_0.05_Fe_2.95_O_4_, reveals the same phenomenon (see Figure S17a,b, Supporting Information). Interestingly, when the NPs were not aligned along the direction of the applied field, magnetic moment alignment still occurred along the NP chain direction, but higher magnetic field strengths were necessary to achieve full alignment with the external field. This likely results from magnetization processes occurring along directions differing from the NPs’ easy axis (see Figure S17c,d, Supporting Information).

**Figure 5:**
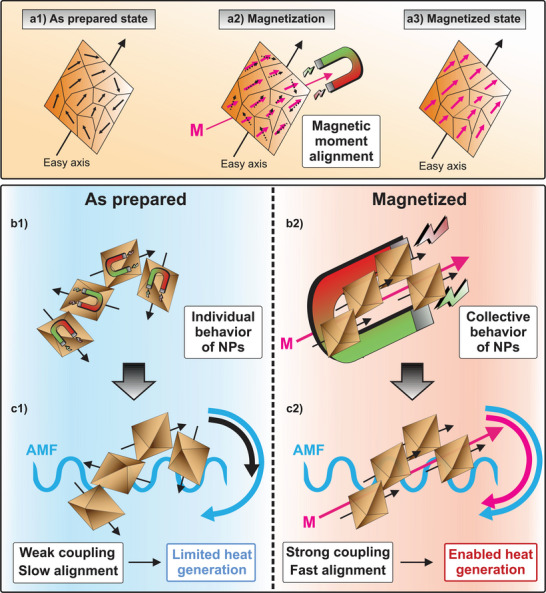
Mechanism of the switchable induction heating behavior in Co‐doped FIONs upon exposure to an external static magnetic field. In the pristine state, the magnetic moments inside the NPs are randomly oriented a1), while pre‐magnetization with a static magnet induces their orientation along the magnetization axis a2). This pre‐aligned state remains once the external magnetic field is removed a3). The difference between the pristine and the magnetized NP state lies in the assumption that while without magnetization, the individual NPs are oriented randomly and behave like individual magnets b1), the magnet‐induced alignment in the magnetized state triggers their collective behavior as a larger magnetic unit b2). Therefore, in the as‐prepared state, heat generation is only limited due to insufficient coupling with an applied AMF c1), while in the magnetized state alignment is induced on a faster scale, causing enhanced induction heating characteristics c2).

Based on these findings, we created a scheme (**Figure** **5**) to visualize the mechanism of the attainment of an improved induction heating performance in Co‐doped FIONs after exposure to an external magnetic field. An enhanced induction heating performance of magnetic NPs after pre‐alignment has also been reported elsewhere in the literature (for a more detailed discussion, see Supporting Information).^[^ ^43^, ^44^, ^45^, ^46^ ^]^ In the NPs’ pristine state, the magnetic moments inside their domains are oriented non‐uniformly, as in this state, no magnetic field was present during their synthesis (Figure 5a‐1). When the NPs are pre‐magnetized with a static magnet, the alignment of magnetic moments along the magnetization axis is induced due to an external magnetic force acting on them (Figure 5a‐2). Upon removal of the static magnetic field, the magnetic alignment within the NPs is retained due to the enhanced anisotropy introduced by Co doping. This leads to a remanent magnetized state that is distinct from the pristine configuration, owing to the preferential orientation of the magnetic moments along the easy axes (Figure 5a‐3). We further hypothesize that the large static magnetic field also “unsticks” frozen spins within the NPs, a phenomenon similar to mechanisms exploited in spin valves,^[^ ^47^ ^]^ which contributes to an increase in magnetic hysteresis and consequently enhances energy dissipation during induction heating.

The difference in the NP behavior in their pristine and magnetized state lies in the alignment of the NPs along the magnetization axis of the external magnet. When no prior magnetization occurs, the Co‐doped FIONs act as individual small magnets with no preferred direction of their magnetic moments (Figure 5b‐1). Hence, by application of an AMF during induction heating, the magnetic field strength provided by the induction heating device (500 Oe) is not sufficient to magnetize the samples and therefore align their magnetic moments. Hence, they do not act as an ensemble, and magnetization reversal is not possible. Instead, all magnetic moments would need to align individually to follow the applied field, which is not feasible in the provided time frame, resulting in weak coupling (Figure 5c‐1). On the other hand, if the NPs are initially aligned by sufficiently strong external magnetization, they do not behave individually, but rather as an ensemble, or so to say, one larger magnetic entity (Figure 5b‐2). This becomes apparent by taking the OAEH data into account, where the chain of investigated NPs behaves like a fused rod magnet once the sample is magnetically saturated. In this state, all magnetic moments are already pre‐aligned, which enables the NPs to follow an applied AMF on a much faster time scale as the additional factor of aligning magnetic moments of individual NPs falls away. This results in strong coupling and higher temperatures reached during the induction heating process (Figure 5c‐2). Remarkably, the NPs’ ability to behave as one unit is only triggered by the external magnetic switch that has to be strong enough to align all NPs to behave collectively. As OAEH data indicates, this is only dependent on the position of the magnetized NPs to the external applied field – if the NP's easy axis is oriented in the same direction as the external field, saturation is reached at lower field strengths compared to a sample situated perpendicular to the external field.

### 2.4 Proof of Principle of Switchable Induction Heating in Application‐Oriented Debonding Scenarios

The enhanced induction heating performance by the alignment of magnetic moments upon pre‐magnetization is characterized not only by its remote and quick character, but especially by the possibility of local triggering. This could be beneficial in use cases where specific items need to be activated for induction heating that are positioned close to others that should not (yet) be switched on. Thus, local magnetic pre‐programming becomes feasible when the spatially resolved nature of external pre‐magnetization is investigated. Note that while the NPs used in powder form were pre‐magnetized before being transferred to the measurement setup, resulting in a random orientation of their magnetic moments, the composites were uniformly magnetized with the static field applied perpendicular to their measured surface, leading to a well‐defined and consistent alignment of magnetic moments.

To explore this, a system is tested in which supraparticles (SPs) of Co_0.05_Fe_2.95_O_4_ are incorporated into a polydimethylsiloxane (PDMS) matrix (30 wt%). For this, SPs are synthesized via spray‐drying, a process where the corresponding dispersion of Co‐doped FIONs is atomized by a nozzle and fed into a hot chamber where the solvent is evaporated. In turn, the individual NP building blocks are forced into micron‐scaled assemblies, so‐called SPs (see Figure S18, Supporting Information).^[^ ^48^ ^]^ Using SPs instead of dried NP agglomerates for integration into matrix environments offers significant advantages, as it effectively prevents the formation of large agglomerates that could lead to an uneven distribution within the matrix. By utilizing the spray‐drying process, a fine powder is produced while minimizing the occurrence of larger clusters (see Figure S19, Supporting Information for SEM of individual SPs as well as a comparison with NP agglomerates).^[^ ^11^ ^]^ Ensuring a uniform distribution of NPs is critical, as uneven dispersal within the matrix can result in inconsistent induction heating performance. SEM images reveal individual SPs incorporated into a PDMS environment that is further confirmed by energy‐dispersive X‐ray (EDX) analysis, indicating Fe and Co on the spots of SPs, while Si is only present in the matrix, demonstrating the successful manufacturing of the composite material (see Figure S20b,c, Supporting Information).

**Figure 6:**
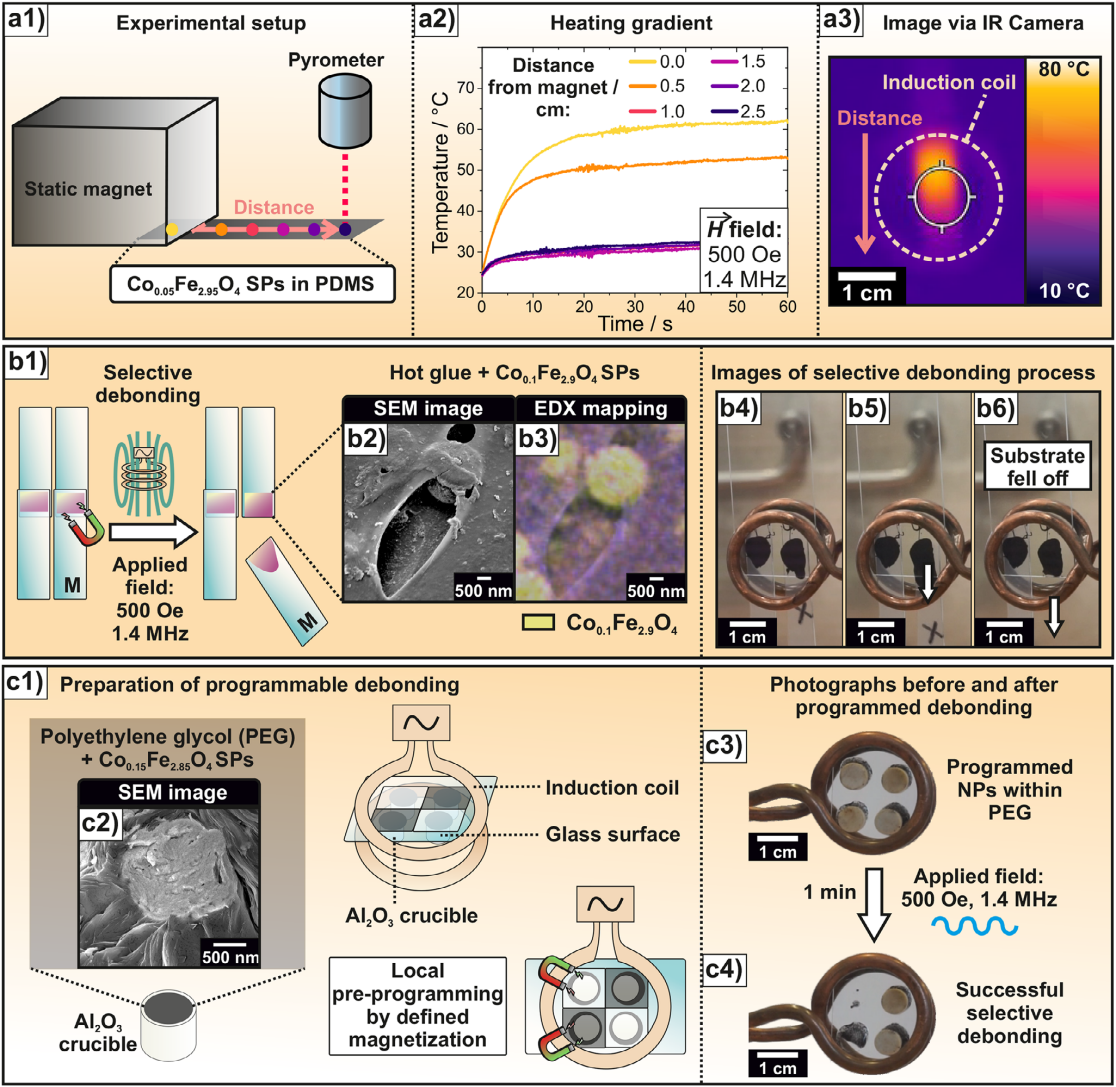
Local programming of induction heating in Co‐doped FIONs and proof of principle of debonding on demand by induction heating. For all induction heating experiments, a field amplitude of 500 Oe and an AMF frequency of 1419 kHz were employed. a) Local pre‐magnetization of a composite of Co_0.05_Fe_2.95_O_4_ SPs incorporated into a PDMS matrix 1). The temperature during induction heating was measured at different distances to the static magnet 2). The data revealed that heat production is only measurable at a distance up to 0.5 cm, indicating the local character of pre‐magnetization that is further supported by imaging the composite with a thermal camera 3). b) Selective debonding on demand demonstrated by attaching two weighted plastic samples to a glass slide and magnetizing only one side (symbolized by M, 1). For this, a composite made of Co_0.1_Fe_2.9_O_4_ SPs incorporated into hot glue is utilized 2, 3). The proof of principle was achieved by selectively debonding only the pre‐magnetized part (marked with an X) during induction heating, while the unmagnetized sample remained attached 4−6). Scale bars represent 1 cm. c) Programmable debonding demonstrated by attaching four Al_2_O_3_ crucibles filled with a composite of Co_0.15_Fe_2.85_O_4_ SPs incorporated into PEG 2) onto a glass slide. By defined local pre‐magnetization, selected crucibles can be programmed toward induction heating 1). Doing so, the pre‐programmed crucibles are debonded during induction heating in ≈1 min 3–4). Scale bars represent 1 cm.

The composite material was positioned under a static magnet (4 kOe), with a portion of the coating concealed by the magnet while leaving the rest uncovered. Marks were made at 0.5 cm intervals along the coating (**Figure** **6**a‐1, see Figure S20a, Supporting Information for photo). The coating was then cut, and temperature measurements were taken at each marked point using a pyrometer during induction heating, including the section under the magnet (Figure 6a‐2). The section under the magnet reached the highest maximum heating temperature during induction heating (62 °C after 60 s). At a distance of 0.5 cm, the temperature was 53 °C, and beyond 1 cm, the temperature stabilized at 32–33 °C, which underlines the local magnetization of Co‐doped FIONs in a surrounding environment below 1 cm. This temperature gradient is further presented by tracking the heat development during induction heating with a thermal imaging camera (Figure 6a‐3), where the part closest to the magnet reaches the highest temperatures (corresponding video is available online, Video S1, Supporting Information).

The local nature of improved induction heating upon pre‐magnetization was further exploited in debonding on demand scenarios, where debonding of a particle‐containing matrix should be triggered by specific external magnetization of a specific part before induction heating, which could be crucial in real‐world recycling scenarios. As a first example, a model system was established in which Co_0.1_Fe_2.9_O_4_ SPs were incorporated into a hot glue matrix (ethylene vinyl acetate copolymer, 20 wt%) that was applied to attach two weighted plastic probes to a glass slide (Figure 6b‐1). SEM with EDX‐analysis revealed the successful incorporation of the corresponding SPs into the surrounding environment (Figure 6b‐2,3; Figure S21a,b, Supporting Information). To demonstrate debonding on demand, only one side was pre‐magnetized (marked with an M in the scheme and X in the experiment; full video is accessible online, Video S2, Supporting Information) and therefore programmed for enhanced induction heating. When the sample is inductively heated with an amplitude of 500 Oe, debonding occurs after ≈90 s specifically on the pre‐magnetized side, while the unmagnetized part remains attached (Figure 6b‐4–6). Corresponding induction heating curves show the magnetized spot heating to 55 °C, whereas the as‐prepared part only heated to 35 °C (see Figure S21c, with SEM images of the incorporated SPs in Figure S21d, Supporting Information). This data indicates the successful improvement of induction heating due to pre‐magnetization in debonding on demand scenarios.

Going one step further, debonding on demand on two adjacent spots can be extended to the targeted pre‐programming of several sites due to the possibility of applying a magnetic field locally. This could simulate debonding scenarios in more complex multi‐component systems. For this means, Co_0.15_Fe_2.85_O_4_ SPs were integrated into a polyethylene glycol (PEG) matrix (20 wt%), before four Al_2_O_3_ crucibles were filled with the composite and attached in the heated state to a glass slide (Figure 6c‐1). SEM imaging revealed the successful integration of the corresponding SPs into PEG (Figure 6c‐2, see Figure S22a,b, Supporting Information for EDX analysis). Subsequently, two spots were locally pre‐programmed toward induction heating by applying a static magnetic field. Upon applying an AMF of 500 Oe, the specific debonding of both pre‐selected crucibles was performed in ≈1 min, while the unmagnetized crucibles remained conjoined (Figure 6c‐3‐4, see full video accessible online, Video S3, Supporting Information). Respective induction heating curves revealed significantly higher temperatures of 60 °C achieved in the pre‐magnetized spots, leading to debonding, as opposed to 45 °C in the untreated sites (see Figure S22c,d, Supporting Information with SEM images of the incorporated SPs). The high induction heating temperatures reached in the investigated time frame, as compared to the other matrix materials, stem from the higher thermal conductivity of the PEG environment. This phenomenon is further discussed in the Supporting Information (see also Figure S23, Supporting Information). The data shows that not only local, but also selective programmable debonding on demand of specifically only magnetized samples is possible by applying the external pre‐magnetization. Most importantly, while all parts are exposed to the applied induction field, explicitly magnetized areas are fully responsive to it, resulting in enhanced heating rates. Therefore, even when an induction field is not well resolved in an application‐oriented scenario, a good resolution can be achieved nonetheless, stemming from precise pre‐programming of incorporated particles. Both model systems act as proof of principles toward debonding on demand, introducing a fast, locally resolved, variably applicable, and selective possibility to optimize particle‐based composites for real‐life recycling scenarios. While our current focus is on more technical implementations involving magnetic fields and frequencies well above those typically allowed for biomedical applications, we have shown that through Co doping, our NPs are highly tunable. This tunability suggests that similar effects could be achieved at lower magnetic field strengths and frequencies compatible with biomedical safety limits.

## 3 Conclusion

In conclusion, we demonstrated the local programming of Co‐doped FIONs toward induction heating through specific pre‐magnetization. By synthesizing six samples of the type Co_X_Fe_3‐X_O_4_ with Co dopings ranging from X = 0.05 to 0.3, we established a clear relationship between the Co content and the NPs’ magnetic properties. Higher Co doping increased the coercivity of the NP samples from 160 to 1080 Oe due to an enhanced anisotropy. In their pristine state, only the three lowest‐doped FIONs effectively produced heat upon induction heating. However, pre‐magnetization with a 4000 Oe static magnetic field allows for programming all samples for enhanced induction heating and improves heat generation in those already triggered in their unmagnetized state. In this manner, an enhanced induction heating performance is achieved without increasing the amount of employed NPs. It was shown that the programmed enhanced induction heating is induced at higher static magnetic field strengths upon higher Co doping. The mechanism behind the switch of the induction heating performance likely lies in the pre‐arrangement of magnetic moments inside the domains along the external magnetization axis upon exposure to a static magnetic field. This alignment remains once the magnet is removed, allowing a faster response to an applied AMF and a stronger coupling as opposed to the undirected state. The pre‐magnetization and therefore enhanced induction heating of Co‐doped FIONs was exploited in debonding on demand scenarios to achieve a proof of principle. It was found that the pre‐magnetization process of particle‐containing composites leads to a localized programming for a later trigger by an AMF. This approach supports not only specific and local debonding on demand in a two‐component system, but also pre‐programmed debonding with more complex set‐ups.

The switchable induction heating properties of the presented NPs, as well as their freely designable magnetic characteristics in terms of the chosen Co doping highlight their potential for advanced applications. As the magnetic alignment introduced by pre‐magnetization remains even after the external magnet is removed, particle‐based composites for industrial recycling processes could be optimized where debonding has to be selectively activated at the end of a product's lifetime. Moreover, by careful adaptation of the presented NPs, use cases could be steered toward the remotely triggerable programming of induction heating in biological tissues in the future, offering possibilities in applications like implant‐based therapies.

## 4 Experimental Section

### Materials and Reagents

Iron(II) sulfate heptahydrate (FeSO_4_·7 H_2_O, 99.5%) and potassium nitrate (KNO_3_, 99+%) were obtained from Acros Organics. Potassium hydroxide (KOH, 90%, flakes) was received from Carl Roth. Cobalt(II) sulfate heptahydrate (CoSO_4_·7 H_2_O, 99+%), manganese(II) sulfate monohydrate (MnSO_4_·H_2_O, ≥98%), nickel(II) sulfate hexahydrate (NiSO_4_·6 H_2_O, ≥98%), and poly(ethylene gycol) (PEG, M_W_ ≈ 10 000) were bought from Sigma Aldrich. Hot glue (ethylene vinyl acetate copolymer, hotmelt sticks) was received from Pattex. Vinyl‐terminated polydimethylsiloxane (PDMS, polymer VS 20 000, vinyl content 0.04 mmol g^−1^), PDMS with SiH groups in the polymer chain (cross‐linker 120, SiH content 1.10 mmol∙g^−1^) and divinyl tetramethyl disiloxane‐platinum(0)‐complex (catalyst 512 in TR50 silicon tenside, Pt content 0.2 wt%) were purchased from Evonik. All chemicals and reagents were used without further purification. Water was deionized before usage.

### Synthesis of Co‐Doped FION NPs

Co‐doped FIONs were synthesized by an oxidative precipitation method formerly introduced by the group^[^ ^27^ ^]^ and subsequently modified by the addition of Co.^[^ ^11^ ^]^ During the synthesis, exposure to magnetic fields was prevented by using an overhead stirrer and a heating mantle.^[^ ^49^ ^]^ For the synthesis of Co_0.3_Fe_2.7_O_4_ NPs, FeSO_4_·7 H_2_O (9.01 g, 32.4 mmol) and CoSO_4_·7 H_2_O (1.01 g, 3.60 mmol) were dissolved in 1620 g of deionized H_2_O and brought to reflux while degassing with nitrogen. In a separate beaker, KNO_3_ (25.48 g, 252.02 mmol) and KOH (6.17 g, 109.96 mmol) were dissolved in 180 g of deionized water to form the precipitating agent while degassing with nitrogen and heating to 60 °C. After reaching the required temperatures, the precipitating agent was added to the reaction solution under a color change to dark brown. The reaction mixture was left to boil for 5 h under reflux cooling and degassing with nitrogen, creating a dark brown suspension. Afterward, the mixture was allowed to cool down to room temperature, before the sedimented NPs were purified by centrifugation (3 × 8000 rpm for 2 min) and redispersed in 100 mL of deionized water to create an aqueous dispersion of Co‐doped FIONs. For other Co dopings, the molar ratios of FeSO_4_·7 H_2_O and CoSO_4_·7 H_2_O were adjusted accordingly. For Ni and Mn dopings, the molar ratio of X_0.3_Fe_2.7_O_4_ was maintained while the corresponding sulfate hydrate salts were used. For undoped species, ratios were adjusted accordingly for Fe_3_O_4_. Pre‐magnetization on selected NP samples was performed post‐synthesis with a static magnet (block magnet 50.8·50.8·25.4 mm, type: NdFeB, magnetization: N40, coating: Ni‐Cu–Ni, maximum operating temperature: 80 °C, adhesive force: 100 kg, magnetic field strength: ≈4000 Oe). Oven heating to the Curie temperature was performed by treating respective dried NPs in a high‐temperature muffle furnace (Nabertherm 5/11 with B400/B410 controller) at 700 °C for 5 min.

### Synthesis of Co‐Doped FION SPs

SPs of Co‐doped FIONs were fabricated by spray‐drying aqueous dispersions of the corresponding NPs using a laboratory‐scale Büchi Labortechnik AG spray‐dryer (B‐290 mini) connected to a dehumidifier B‐296. For all spray‐drying procedures, the weight concentration of the dispersions was maintained at 2.7 wt% and adapted as required using deionized H_2_O. All runs were conducted using identical technical parameters (inlet temperature: 85 °C, pump rate: ≈0.18 L h^−1^, aspirator power: 85%, resulting in a volume flow of ∼ 33 m^3^ h^−1^, spraying gas flow: 473 L h^−1^, outlet temperature: ≈40 °C).

### Fabrication of Co‐Doped FION/PDMS Composites and Test of Spatially Resolved Induction Heating

The composite was prepared from Co_0.05_Fe_2.95_O_4_ SPs (0.3 g), vinyl terminated PDMS (0.671 g, polymer VS 20.000), and PDMS with SiH groups (0.029 g, cross‐linker 120) by mixing all components with a gravity mixer (SpeedMixerTM DAC 150 1 FVZ Hausschild) for 2 min at 3500 rpm. Two droplets of divinyl tetramethyl disiloxane‐platinum(0)‐complex (catalyst 512) were added to the composite mixture and mixed for 30 s at 3500 rpm with the gravity mixer. The resulting viscous mixture was transferred onto a polyethylene foil (125 µm thickness), and a coated film was generated using a coating blade (MULTICATOR 411, adjusted to 100 µm) and a film applicator (COATMASTER 510, speed setting 10). Afterward, the mixture was cured for 2 h at 100 °C to initiate the cross‐linking of the composite. To assess the spatial resolution, a static magnet (NdFeB, coating: Ni–Cu–Ni, adhesive force: 100 kg, magnetic field strength: ≈ 4000 Oe) was partially placed over the coating, leaving the other part of the coating exposed. Marks were made at 0.5 cm intervals along the coating. The coating was then cut, and measurements were taken at the marked points, including the section under the magnet. With the locally magnetized composite, the gradient of locally resolved induction heating was tested by placing the material inside a two‐wind coil (Cu, frequency: 1419 kHz) and applying a magnetic field with an amplitude of 500 Oe. Simultaneously, heat production was monitored using a thermal imaging camera (FLIR C5, Teledyne Flir).

### Fabrication of Composites and Test of Programmable Debonding

Composites of Co_0.15_Fe_2.85_O_4_ SPs and PEG (20 wt%) were prepared by mixing their respective powders in a gravity mixer (SpeedMixer DAC 150.1 FVZ, Hauschild) at 3500 rpm for 3 min. The resulting mixture was transferred into 75 µL Al_2_O_3_ crucibles, with four samples heated to 100 °C in an oven and subsequently affixed to a glass slide. Two of the samples were magnetized before positioning the glass slide within a two‐wind copper coil (frequency: 1419 kHz). A magnetic field with an amplitude of 500 Oe was then applied using the induction heating device.

Composites of Co_0.05_Fe_2.95_O_4_ SPs in hot glue were created by combining the SPs (0.2 g) with hot glue (0.8 g) in a ratio of 1:4. The mixture was heated to 150 °C for 1 h, before optimal mixing was induced by mechanical stirring. For the application‐oriented debonding scenario, the composites were applied to a glass slide in the heated and therefore viscous state, after which two plastic parts were glued onto it. After they were allowed to cool down to room temperature, the plastic parts were weighted with two water‐filled glass vials. Debonding was triggered in the pre‐magnetized sample (magnet: NdFeB, coating: Ni‐Cu‐Ni, adhesive force: 100 kg, magnetic field strength: ∼ 4000 Oe) upon exposure to an AMF with an amplitude of 500 Oe.

### Material characterization

Scanning electron microscopy (SEM) characterization was conducted using a JSM‐F100 (Jeol). Powder‐based NP and SP samples were prepared on carbon pads (Plano), while composite samples were cut with a razor blade. All samples were sputtered with Pt (108SE, Cressington, 40 mA, 20 s). Morphology was examined using a secondary electron detector at a working distance of 6 mm with an acceleration voltage of 10 kV for powder samples and 5 kV for composites, respectively. EDX analyses were conducted with an acceleration voltage of 15 kV and a working distance of 10 mm.

Induction heating experiments were conducted employing the high‐frequency device Sinus 52 (Himmelwerk) with a two‐wind coil (Cu) at a frequency of 1419 kHz and AC magnetic fields with a maximum amplitude of 500 Oe. The magnetic field amplitudes were simulated in collaboration with the Himmelwerk company. The temperature at the surface of magnetic samples was measured with a CTlaser LTF pyrometer (Optris). The magnetic field amplitude was adjusted manually to different values (50 to 500 Oe) depending on the experiment. For the induction heating of dried NP powder samples, 30 mg of the respective sample was transferred to a 75 µL crucible (Al_2_O_3_). For both powders and application‐based composites, the corresponding materials were placed in the center of the coil. Some induction heating measurements were performed on magnetized samples. To prepare these, the powders were exposed to a static magnetic field by placing a permanent magnet (NdFeB, coating: Ni–Cu–Ni, adhesive force: 100 kg, magnetic field strength: ≈4000 Oe) beneath the vial containing the powder before heating experiments. After magnetization, the powder was transferred to the crucible, resulting in a random orientation of NPs for the applied AMF. No deliberate control of the angle between the initial magnetizing field and the AC magnetic field was applied during heating measurements.

Magnetization curves of magnetic NP samples were obtained using a superconducting quantum interference device (SQUID) MPMS3 (Quantum Design Inc.). Static magnetization curves to approximate the alternating magnetic field present in the induction heating device were recorded by cycling the applied field from −500 to 500 Oe with a step rate of 2 Oe s^−1^. Larger magnetization curves were obtained by cycling the applied magnetic field from −30 to 30 kOe employing a step rate of 50 Oe s^−1^. The temperature for all measurements was set to 300 K except explicitly stated otherwise. For experiments during which the sample was magnetized to a specific value before measuring the magnetization curve, the corresponding magnetic field was reached in the device starting from an unmagnetized sample and employing a linear approach with a step rate of 50 Oe s^−1^. AC magnetization curves were recorded by cycling the applied field from −500 to 500 Oe with an amplitude of 3 Oe and a frequency of 1000 Hz.

Transmission electron microscopy (TEM) images were recorded with a LEO EM 900 microscope (Carl Zeiss AG, Oberkochen, Germany) using an acceleration voltage of 80 kV. NP size distributions were averaged over 75 NPs from TEM images by manual evaluation using ImageJ.

Thermogravimetric analysis (TGA) measurements for Curie temperature determination were conducted on 3–10 mg of dried, pre‐magnetized NPs with an attached static high‐energy magnet (Netzsch, TGA209F1E93.100‐00) on a TG 209 F1 Libra (Netzsch) by heating the samples in a nitrogen atmosphere (N_2_: 50 mL min^−1^) with a heating rate of 30 K min^−1^ in the temperature range from 30 to 1000 °C. For a more reliable measurement, a non‐magnetic counterweight in the form of SiO_2_ (sand, Carl Roth) was utilized to prevent mass falsifications induced by the lifting of the crucible lid, which is otherwise caused by the experimental setup.

The crystallinity of the samples was analyzed via X‐ray diffraction (XRD) using a D8 Advance diffractometer (Bruker) equipped with a Lynxeye XE‐T detector employing Cu Kα radiation (*λ* = 0.154056 nm). Measurements were conducted in the 2θ range from 20 to 80° at 0.02° increments. Indication of reflections was performed based on the International Centre for Diffraction Data PDF‐4.

The Co content in respective Co‐doped FION species was determined by inductively coupled plasma atomic emission spectroscopy (ICP‐AES) using a Ciros CCD (Spectro Analytical Instruments GmbH) and an Optima 8300 (PerkinElmer). The solid samples were dissolved in concentrated HCl:HNO_3_:HF in a volumetric ratio of 3:1:1 in 100 mL, employing microwave heating to 220 °C for 40 min.

Off‐axis electron holography was performed in an image‐Cs‐corrected FEI Titan 80–300 microscope equipped with two electron biprisms at 300 kV. A homemade in‐plane rotation TEM holder was used. Off‐axis electron holograms were recorded in magnetic field‐free conditions using a direct electron‐counting camera (Gatan K2 IS). The phase shift of an electron wave was reconstructed from holograms resulting from the presence of magnetic and electric fields. To separate the two contributions of the phase shift, the specimen was tilted to ±79° and the magnetic field of the conventional objective lens of the microscope was used to magnetize it in opposite directions in a field of about 1.5 T. Half of the sum and half of the difference between aligned phase images reconstructed from such pairs of off‐axis electron holograms were used to determine the electrostatic and magnetic contributions to the phase, respectively. The electrostatic contribution to the phase is based on the mean inner and electrostatic potentials of the specimen integrated in the electron beam direction, while the gradient of the magnetic contribution to the phase is proportional to the strength and direction of the in‐plane magnetic induction projected in the electron beam direction. The corresponding NPs were magnetized along the chain axis to specific values, at which the mean inner potential component was reconstructed, and magnetic phase images were converted into magnetic contour images.

Videos were recorded with a digital camera (Alpha6000, Sony), while snapshots of the videos were taken at characteristic times during the induction heating procedure. Digital photographs were taken with a Redmi Note 13 Xiaomi (2024).

## Conflict of Interest

The authors declare no conflict of interest.

## Author Contributions

T.R. and L.L. contributed equally to this work. T.R. led the conceptualization, investigation, methodology, data curation, supervision, validation, visualization, original draft writing, and wrote, reviewed, and edited the manuscript. L.L. also played a leading role in conceptualization, investigation, methodology, data curation, supervision, validation, visualization, and both the original drafting and reviewing/editing of the manuscript, and contributed equally to funding acquisition. S.M. contributed equally to conceptualization, methodology, supervision, visualization, and reviewed and edited the final manuscript. N.K. participated equally in conceptualization, methodology, and reviewed and edited the final manuscript. Q.L. and T.D. contributed equally to the investigation and reviewed and edited the final manuscript. R.E.D.‐B. was involved in the investigation and reviewed and edited the final manuscript. K.M. had a leading role in conceptualization, funding acquisition, project administration, and resource provision, and contributed equally to supervision and reviewed and edited the final manuscript.

## Supporting information



Supporting Information

Supplemental Video 1

Supplemental Video 2

Supplemental Video 3

## Data Availability

The data that support the findings of this study are openly available on Zenodo 10.5281/zenodo.16308522.

## References

[1] E. C. Abenojar , S. Wickramasinghe , J. Bas‐Concepcion , A. C. S. Samia , Prog. Nat. Sci.: Mater. Int. 2016, 26, 440.

[2] a) O. Lucia , P. Maussion , E. J. Dede , J. M. Burdio , IEEE Trans. Ind. Electron. 2014, 61, 2509;

[3] a) H. Caglar , Y. A. Aksoy , S. Idapalapati , B. Caglar , M. Sharma , C. K. Sin , J. Adhes. 2024, 100, 734;

[4] a) M. R. Almind , M. G. Vinum , S. T. Wismann , M. F. Hansen , S. B. Vendelbo , J. S. Engbæk , P. M. Mortensen , I. Chorkendorff , C. Frandsen , ACS Appl. Nano Mater 2021, 4, 11537;

[5] S. R. Yassine , Z. Fatfat , G. H. Darwish , P. Karam , Catal. Sci. Technol. 2020, 10, 3890.

[6] a) W. Fan , Y. Zhang , W. Li , W. Wang , X. Zhao , L. Song , Chem. Eng. J. 2019, 368, 1033;

[7] a) A. Adogwa , E. Chukwu , A. Malaj , V. R. Punyapu , O. Chamness , N. Glisson , B. Bruce , S. Lee , M. J. Zachman , D. A. Bruce , R. B. Getman , O. T. Mefford , M. Yang , ACS Catal. 2024, 14, 4008;

[8] R. Chen , M. G. Christiansen , P. Anikeeva , ACS Nano 2013, 7, 8990. 24016039 10.1021/nn4035266

[9] M. Mündlein , B. Schug , S. Wintzheimer , K. Mandel , J. Magn. Magn. Mater. 2019, 488, 165350.

[10] N. D. a. Silva Moura , K. R. Bajgiran , A. T. Melvin , K. M. Dooley , J. A. Dorman , ACS Appl. Nano Mater. 2021, 4, 13778. 10.1021/acsanm.1c04351 PMC8961733 35372795

[11] T. Raczka , A. Wolf , J. Reichstein , C. Stauch , B. Schug , S. Müssig , K. Mandel , J. Magn. Magn. Mater. 2024, 598, 172042.

[12] J. Reichstein , T. Raczka , C. Stauch , B. Schug , S. Müssig , K. Mandel , Adv. Eng. Mater. 2024, 26, 2400744.

[13] J. Mohapatra , F. Zeng , K. Elkins , M. Xing , M. Ghimire , S. Yoon , S. R. Mishra , J. P. Liu , Phys. Chem. Chem. Phys. 2018, 20, 12879. 29700525 10.1039/c7cp08631h

[14] S. Tong , C. A. Quinto , L. Zhang , P. Mohindra , G. Bao , ACS Nano 2017, 11, 6808. 28625045 10.1021/acsnano.7b01762

[15] L. Luthardt , T. Raczka , K. Hurle , S. Müssig , K. Mandel , Adv. Funct. Mater. 2025, 35, 2412296.

[16] R. Yang , X. Yu , H. Li , C. Wang , C. Wu , W. Zhang , W. Guo , J. Alloys Compd. 2021, 851, 156907.

[17] a) J. G. Ovejero , D. Cabrera , J. Carrey , T. Valdivielso , G. Salas , F. J. Teran , Phys. Chem. Chem. Phys. 2016, 18, 10954; 27041536 10.1039/c6cp00468g

[18] X. K. Zhang , Y. F. Li , J. Q. Xiao , E. D. Wetzel , J. Appl. Phys. 2003, 93, 7124.

[19] P. Caetano , A. S. Albuquerque , L. E. Fernandez‐Outon , W. Macedo , J. D. Ardisson , J. Alloys Compd. 2018, 758, 247.

[20] A. Salokhe , A. Koli , V. Jadhav , S. Mane‐Gavade , A. Supale , R. Dhabbe , X.‐Y. Yu , S. Sabale , SN Appl. Sci. 2020, 2.

[21] M. B. Kulkarni , Yashas, R. V. , Appl. Mater. Today 2024, 38, 102225.

[22] Z. Liu , F. Yan , Adv. Sci. 2022, 9, 2200264. 10.1002/advs.202200264 PMC9036041 35233988

[23] J. Moon , M. G. Christiansen , S. Rao , C. Marcus , D. C. Bono , D. Rosenfeld , D. Gregurec , G. Varnavides , P.‐H. Chiang , S. Park , P. Anikeeva , Adv. Funct. Mater. 2020, 30, 2000577. 35531589 10.1002/adfm.202000577 PMC9075680

[24] M. R. Barati , C. Selomulya , K. G. Sandeman , K. Suzuki , Appl. Phys. Lett. 2014, 105, 162412.

[25] R. Ghosh , L. Pradhan , Y. P. Devi , S. S. Meena , R. Tewari , A. Kumar , S. Sharma , N. S. Gajbhiye , R. K. Vatsa , B. N. Pandey , R. S. Ningthoujam , J. Mater. Chem. 2011, 21, 13388.

[26] Á. Raya‐Barón , S. Ghosh , J. Mazarío , V. Varela‐Izquierdo , P.‐F. Fazzini , S. Tricard , J. Esvan , B. Chaudret , Mater. Horiz. 2023, 10, 4952. 37609955 10.1039/d3mh00908d

[27] T. Granath , P. Löbmann , K. Mandel , Part. Part. Syst. Charact. 2021, 38, 2000307.

[28] E. Fantechi , C. Innocenti , M. Albino , E. Lottini , C. Sangregorio , J. Magn. Magn. Mater. 2015, 380, 365.

[29] L. T. H. Phong , D. H. Manh , P. H. Nam , V. D. Lam , B. X. Khuyen , B. S. Tung , T. N. Bach , D. K. Tung , N. X. Phuc , T. V. Hung , T. L. Mai , T.‐L. Phan , M. H. Phan , RSC Adv. 2021, 12, 698. 35425141 10.1039/d1ra07407e PMC8978697

[30] Z. E. Gahrouei , S. Labbaf , A. Kermanpur , Phys. E: Low‐Dimens. Syst. Nanostructures 2020, 116, 113759.

[31] M. E. Fleet , J. Solid State Chem. 1986, 62, 75.

[32] S. Anjum , R. Tufail , K. Rashid , R. Zia , S. Riaz , J. Magn. Magn. Mater. 2017, 432, 198.

[33] J. M. Byrne , V. S. Coker , S. Moise , P. L. Wincott , D. J. Vaughan , F. Tuna , E. Arenholz , G. van der Laan , R. A. D. Pattrick , J. R. Lloyd , N. D. Telling , J. R. Soc. Interface 2013, 10, 20130134. 23594814 10.1098/rsif.2013.0134 PMC3645421

[34] a) D. Li , H. Yun , B. T. Diroll , V. V. T. Doan‐Nguyen , J. M. Kikkawa , C. B. Murray , Chem. Mater. 2016, 28, 480;

[35] A. R. Yasemian , M. Almasi Kashi , A. Ramazani , Mater. Res. Express 2020, 7, 016113.

[36] a) R. E. Rosensweig , J. Magn. Magn. Mater. 2002, 252, 370;

[37] J. Mohapatra , M. Xing , J. Beatty , J. Elkins , T. Seda , S. R. Mishra , J. P. Liu , Nanotechnol 2020, 31, 275706. 10.1088/1361-6528/ab84a3 32224519

[38] Q. A. Pankhurst , N. T. Thanh , S. K. Jones , J. Dobson , J. Phys. D: Appl. Phys. 2009, 42, 224001.

[39] C. de Julián Fernández , Phys. Rev. B 2005, 72, 054438.

[40] S. Yoon , J. Magn. Magn. Mater. 2012, 324, 2620.

[41] S. Yoon , K. M. Krishnan , J. Appl. Phys. 2011, 109, 07B534.

[42] P. B. Balakrishnan , N. Silvestri , T. Fernandez‐Cabada , F. Marinaro , S. Fernandes , S. Fiorito , M. Miscuglio , D. Serantes , S. Ruta , K. Livesey , O. Hovorka , R. Chantrell , T. Pellegrino , Adv. Mater. 2020, 32, 2003712. 10.1002/adma.202003712 33002227

[43] M. M. Beck , C. Lammel , B. Gleich , J. Magn. Magn. Mater. 2017, 427, 195.

[44] Z. Sha , X. Cheng , A. D. Charles , Y. Zhou , M. S. Islam , A. N. Rider , S. Peng , M. Lim , V. Timchenko , C. H. Wang , Compos. Struct. 2023, 321, 117304.

[45] I. Conde‐Leborán , D. Serantes , D. Baldomir , J. Magn. Magn. Mater. 2015, 380, 321.

[46] Y. Yang , X. Liu , Y. Lv , T. S. Herng , X. Xu , W. Xia , T. Zhang , J. Fang , W. Xiao , J. Ding , Adv. Funct. Mater. 2015, 25, 812.

[47] a) Q. K. Ong , X.‐M. Lin , A. Wei , J. Phys. Chem. C 2011, 115, 2665; 10.1021/jp110716g PMC3037546 21321674

[48] S. Wintzheimer , L. Luthardt , K. A. Le Cao , I. Imaz , D. Maspoch , T. Ogi , A. Bück , D. P. Debecker , M. Faustini , K. Mandel , Adv. Mater. 2023, 35, 2306648. 10.1002/adma.202306648 37840431

[49] a) J. J. Brunner , M. Krumova , H. Cölfen , E. V. Sturm Née Rosseeva , Beilstein J. Nanotechnol. 2019, 10, 894; 31165016 10.3762/bjnano.10.90 PMC6541330

