# Supplementary material for: Magnetically “Programming” Cobalt‐Doped Iron Oxide Nanoparticles for Localized Induction Heating: Triggering a Collective Effect of Magnetic Moment Alignment on Demand
Source: Adv Mater. 2025 Jul 26;37(40):e07158. doi: 10.1002/adma.202507158 (PMC12510280; doi:10.1002/adma.202507158)
Supplement: Supplementary file 1 — Supporting Information [file ADMA-37-e07158-s003.docx]

# Supporting Information

Magnetically “Programming” Cobalt-Doped Iron Oxide Nanoparticles For Localized Induction Heating: Triggering a Collective Effect of Magnetic Moment Alignment On Demand

Theodor Raczka^†^, Leoni Luthardt^†^, Stephan Müssig, Noah Kent, Qianqian Lan, Thibaud Denneulin, Rafal E. Dunin-Borkowski, and Karl Mandel*

Table S1. ICP-AES measurement data for the Co content of three exemplary Co-doped FION species. The data reveals fitting relations between theoretically calculated and practically recorded values.

|  | **Co_0.05_Fe_2.95_O_4_** | **Co_0.15_Fe_2.85_O_4_** | **Co_0.3_Fe_2.7_O_4_** |
| --- | --- | --- | --- |
| Co content 1 (wt%) | 1.15 | 3.62 | 7.42 |
| Co content 2 (wt%) | 1.14 | 3.56 | 7.44 |
| Co content 3 (wt%) | 1.13 | 3.61 | 7.53 |
| Fe content 1 (wt%) | 90.69 | 59.33 | 55.52 |
| Fe content 2 (wt%) | 92.15 | 59.23 | 55.05 |
| Fe content 3 (wt%) | 91.01 | 60.03 | 55.14 |
| **Mean Co content (wt%)** | **1.14** | **3.60** | **7.46** |
| Theoretical Co content (wt%) | 1.27 | 3.81 | 7.61 |
| **Ratio Co/Fe** | **1.95** | **6.04** | **13.64** |
| Theoretical ratio Co/Fe | 1.69 | 5.26 | 11.11 |


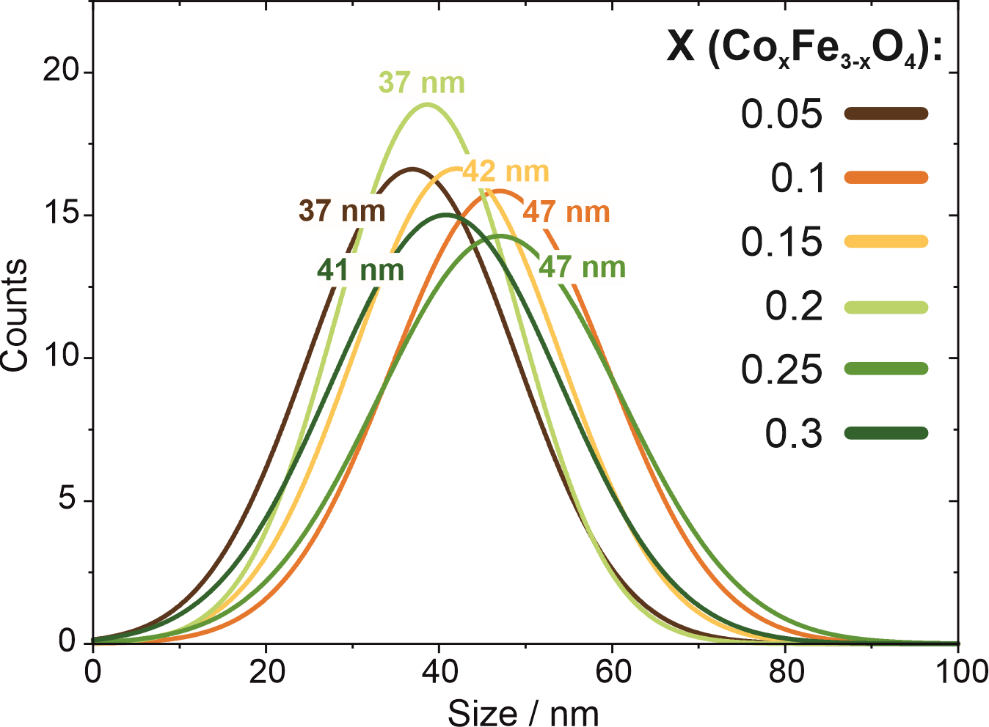


Figure S1. Size distribution plots obtained for all Co-doped FIONs with mean size written out for every NP type. All NP species display a certain degree of polydispersity, however, all reveal comparable mean sizes of approximately 40 nm. Values were obtained from measuring the size of 50 individual NPs from TEM images for every type, respectively.


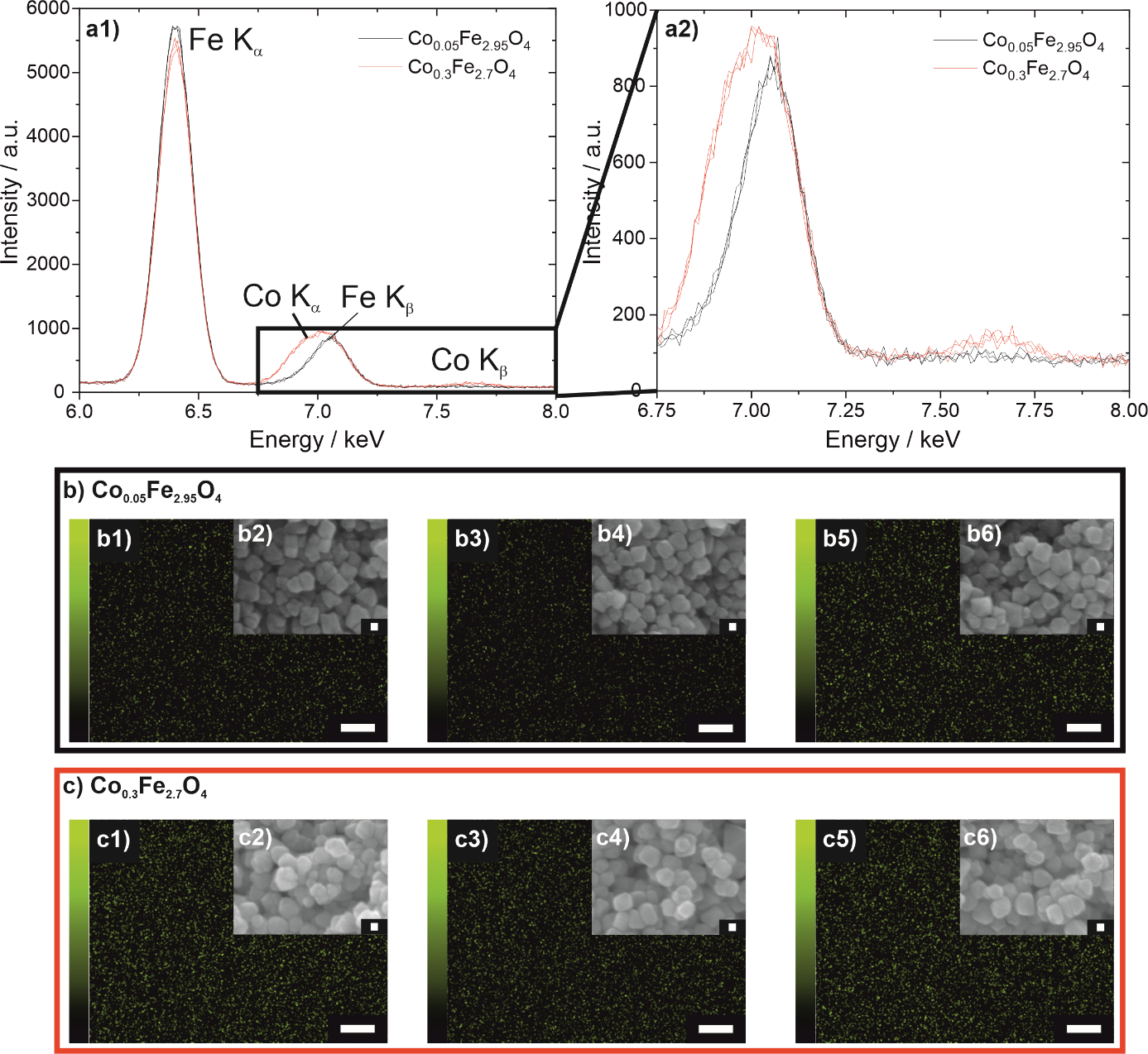


Figure S2. EDX-SEM of Co-doped FIONs. a) EDX spectra indicating a uniform Co and Fe distribution at multiple spots of nanoparticles doped with a different amount. Quantitative count maps of Co in Co_0.05_Fe_2.95_O_4_ (b) and Co_0.3_Fe_2.7_O_4_ (c) taken at three individually different spots conform uniform Co distribution. Scale bars in b1, b3, b5, c1, c3, and c5 represent 50 nm, those in b2, b4, b6, c2, c4, and c6 represent 20 nm.

**Discussion of the XRD diffractograms of Co-doped FIONs**

In the inverse spinel structure of magnetite, Fe^3+^ ions occupy tetrahedral sites, while octahedral sites are inhabited by both Fe^2+^ and Fe^3+^ species.^[1]^ When the Co doping in the magnetite structure is increasing, divalent Co^2+^ replace Fe^2+^ ions and therefore occupy octahedral sites.^[2]^ As the ionic radius of Co^2+^ is smaller compared to that of Fe^2+^, the interplane distances in the spinel structure are increased, thus resulting in the observed reflex shift to lower angles with higher Co doping.^[2,3]^


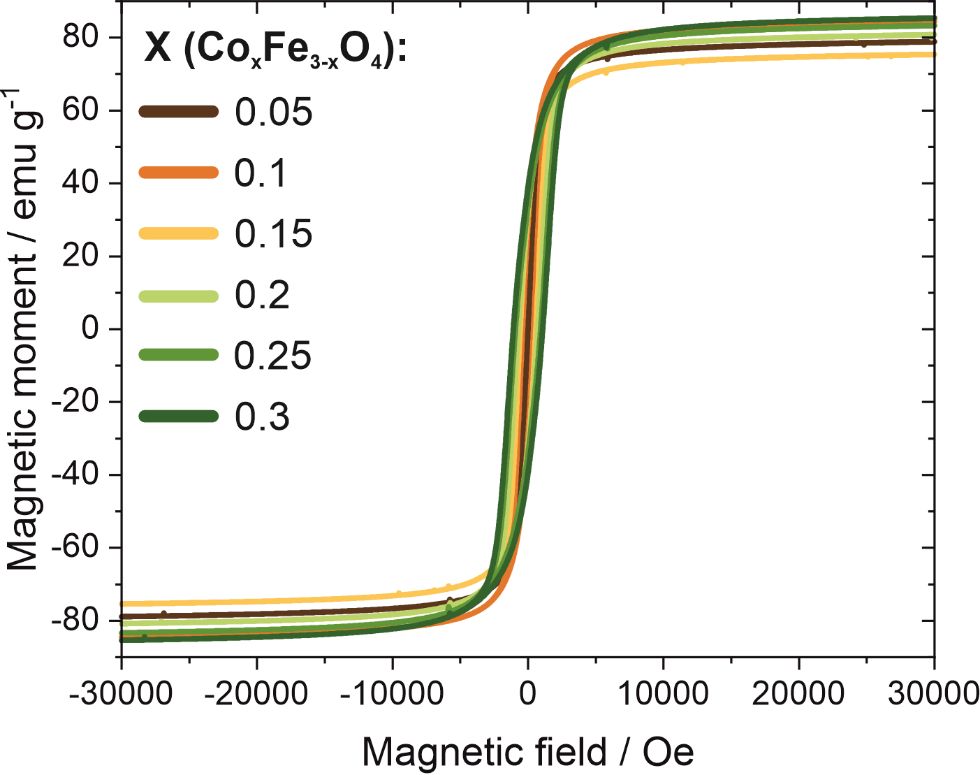


Figure S3. Static (DC) VSM curves for all Co-doped FIONs recorded from -30  to 30 kOe. While the saturation magnetization for all samples stays approximately the same around 80 emu g^‑1^, the coercivity increases with increasing Co doping.

Table S2. Magnetic characterization data obtained from static (DC) VSM measurements of six different Co-doped FIONs of the type Co_x_Fe_3-x_O_4_, with values for coercivity, saturation magnetization, and the anisotropy constant *K* displayed. The unit erg g^‑1^ corresponds to Oe emu g^‑1^.

| **X =** | **0.05** | **0.1** | **0.15** | **0.2** | **0.25** | **0.3** |
| --- | --- | --- | --- | --- | --- | --- |
| **Coercivity / Oe** | 160 | 400 | 575 | 720 | 935 | 1080 |
| **Saturation magnetization / emu g^‑1^** | 79 | 84 | 75 | 81 | 83 | 85 |
| **Anisotropy constant *K* / erg g^-1^** | 13 167 | 35 000 | 44 922 | 60 750 | 80 839 | 95 625 |

**Discussion of the magnetic characterization obtained from static (DC) VSM measurements of Co-doped FIONs**

To contextualize the magnetic properties of our Co_X_Fe_3-X_O_4_ NPs, we compared our results with literature values for similarly sized and doped systems. While precise matches in terms of Co content, morphology, and particle size are limited, several studies report comparable ranges. For example, 40 nm cubic Co_0.1_Fe_2.9_O_4_ NPs show coercivity values around 400 Oe and a saturation magnetization of ≈ 62 emu g^-1^,^[4]^ which aligns reasonably well with our Co_0.1_ sample (Hc ≈ 400 Oe, Ms ≈ 84 emu g^-1^). Similarly, Co_0.3_Fe_2.7_O_4_ NPs with a smaller diameter of ∼16 nm exhibit lower values (Hc ≈ 232 Oe, Ms ≈ 55 emu g^-1^, K ≈ 12 899 erg g^-1^),^[3]^ which is consistent with the expected decrease of magnetic parameters at reduced particle size.

A higher-doped reference (Co_0.6-0.7_, ∼40 nm) shows markedly higher coercivity (Hc ≈ 10.2 kOe) and comparable Ms ≈ 88 emu g^-1^,^[5]^ confirming the trend of increasing anisotropy with Co content. Additional data for smaller NPs supports this picture: 16 nm Co_0.3_Fe_2.7_O_4_ NPs report Ms ≈ 55 emu g^-1^, Hc ≈ 232 Oe, and K ≈ 12 899 erg g^-1^,^[3]^ while Co_0.2_Fe_2.7_O_4_ NPs with a diameter of ∼21 nm exhibit significantly lower anisotropy constants (K ≈ 3 329 erg g^-1^),^[6]^ further underlining the strong influence of both size and composition on magnetic anisotropy.

Our own measurements for Co_X_Fe_3-X_O_4_ NPs (X = 0.05 to 0.3) are in excellent agreement with these trends, showing an increase in coercivity (from 160 Oe for Co_0.05_ to 1080 Oe for Co_0.3_) and saturation magnetization (ranging from 79 to 85 emu g^-1^). Our anisotropy constants (K) also exhibit a marked increase with Co content, from 13 167 erg g^-1^ for Co_0.05_ to 95 625 erg g^-1^ for Co_0.3_, confirming the expected behavior as the cobalt concentration increases.

Overall, our experimentally determined values for coercivity, saturation magnetization, and anisotropy constant fall within or above the reported range for comparable cobalt ferrite systems, supporting the high magnetic quality of the synthesized nanoparticles.


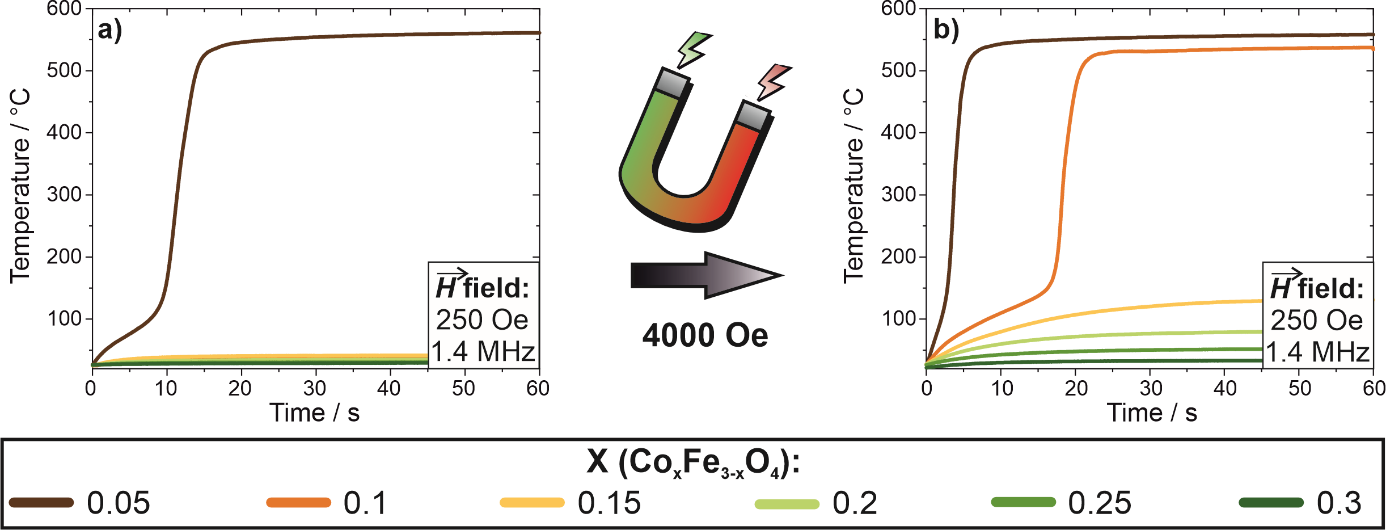


Figure S4. Induction heating curves of all Co-doped FIONs recorded at an AMF amplitude of 250 Oe and an AMF frequency of 1419 kHz before (a) and after (b) pre-magnetization. For all samples, the induction heating behavior is enhanced after prior exposure to a static magnetic field, especially when considering that the NPs with X = 0.1 do not effectively produce heat before magnetization, however, they do afterwards. Despite producing no sufficient thermal energy to initiate induction heating, the other NP species with higher amounts of Co are also enhanced in their induction heating performance.


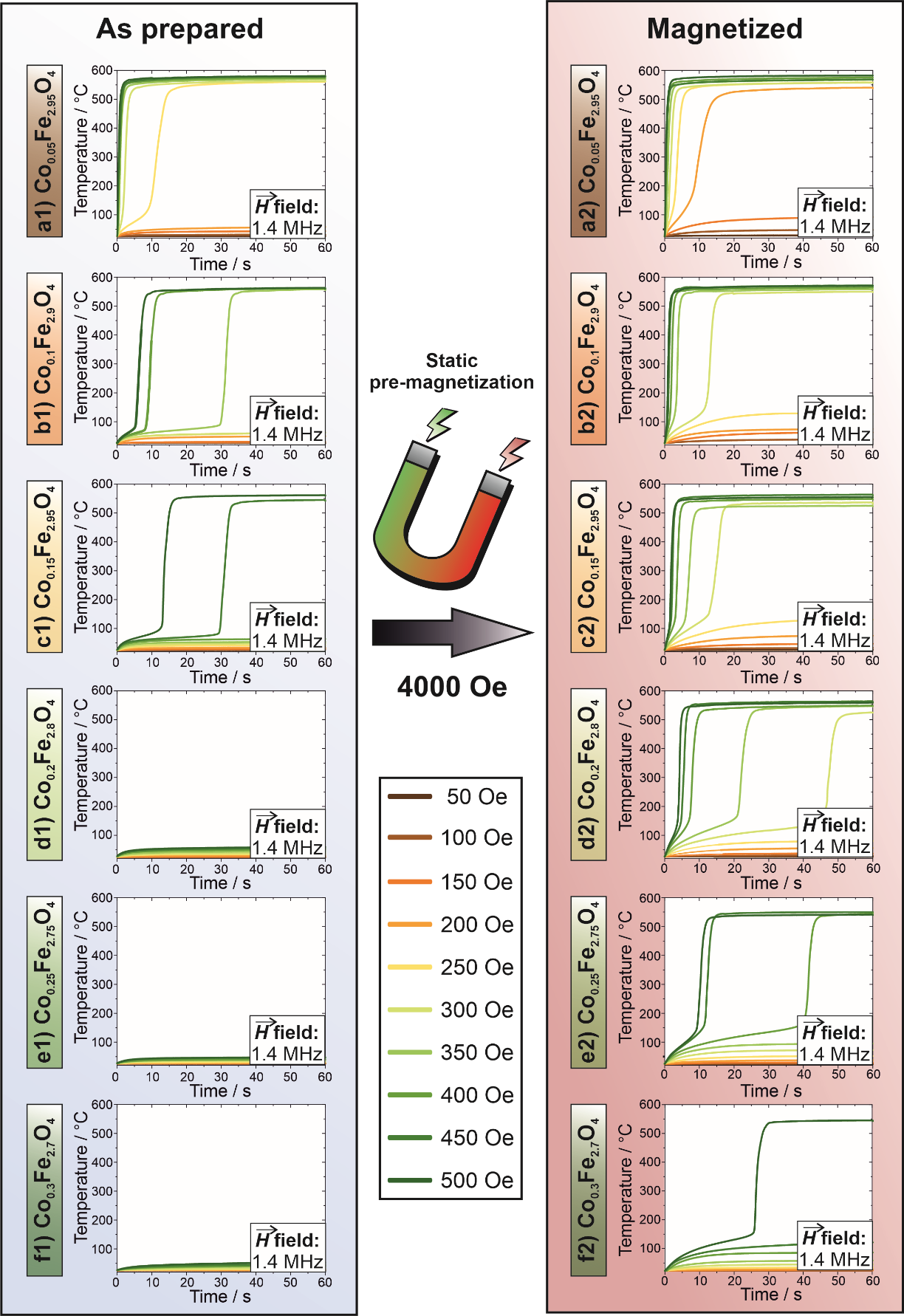


Figure S5. Induction heating curves at an AMF frequency of 1419 kHz and different AMF amplitudes between 50 and 500 Oe for Co-doped FIONs of the type Co_x_Fe_3-x_O_4_ with X = 0.05 (a), 0.1 (b), 0.15 (c), 0.2 (d), 0.25 (e), and 0.3 (f) before (1) and after (2) pre-magnetization. For all NP types, the enhanced induction heating occurs at AMFs with lower amplitudes after prior magnetization compared to the pristine state, underlining the easier magnetizability of Co-doped FIONs after exposure to an external static magnetic field.

Table S3. Coercivity and saturation magnetization values obtained from static (DC) VSM measurements between -500 and 500 Oe for Co_x_Fe_3-x_O_4_ with X = 0.05 to 0.3. Note that, while when recording VSM curves for these samples in a larger static field range, the coercivity is increasing with increasing Co doping, the trend for the minor curves recorded here is opposing. The reason for that is that the hysteresis curves cannot be fully recorded in the given small field, hence the smaller coercivity value here indicates that the respective NP species is not fully magnetizable.

|  | **Coercivity / Oe** | | **Saturation magnetization / emu g^‑1^** | |
| --- | --- | --- | --- | --- |
| **X** | **Before magnetization** | **After magnetization** | **Before magnetization** | **After magnetization** |
| **0.05** | 122 | 100 | 34 | 31 |
| **0.1** | 23 | 134 | 12 | 18 |
| **0.15** | 15 | 111 | 9 | 15 |
| **0.2** | 13 | 47 | 7 | 10 |
| **0.25** | 7 | 83 | 6 | 9 |
| **0.3** | 5 | 102 | 3 | 5 |

**Discussion of the hysteresis curve area of Co_0.05_Fe_2.95_O_4_ before and after pre-magnetization**

For Co_0.05_Fe_2.95_O_4_, the hysteresis curve area does not increase after pre-magnetization, but displays a minor deterioration. When considering the VSM data before and after magnetization (**Figure S5a**), the hysteresis loops seem approximately comparable, however the area is decreased. This is in accordance with a slightly reduced heating rate when considering the induction heating data, manifesting itself in 520 K s^‑1^ for the unmagnetized and 480 K s^‑1^ for the magnetized state. The hypothesis for this behavior is based on the fact that the coercivity of NPs is reduced once they become hot. This effect is facilitated with a pre-magnetization due to an enhanced magnetizability. However, for Co_0.05_Fe_2.95_O_4_, the coercivity in the unmagnetized state is ideal to be optimally excited by the employed induction heating device. If the sample is pre-magnetized, the reduction of the coercivity happens so fast that the induction heating performance is diminished, as a smaller coercivity also corresponds to a decreased hysteresis area.


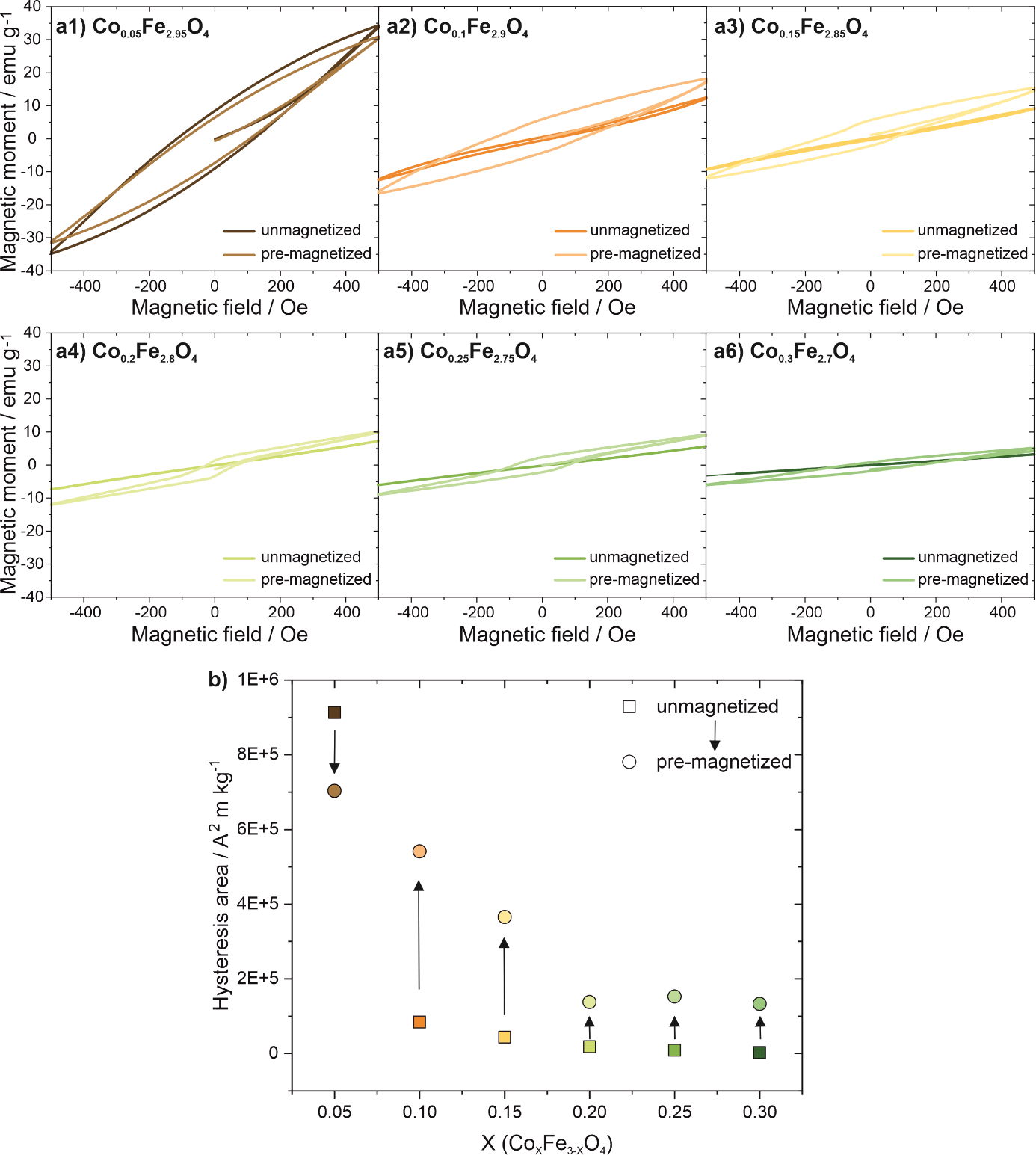


Figure S6. a) Direct comparison of VSM curves of Co-doped FIONs before and after pre-magnetization, recorded between -500 and 500 Oe for NPs of the type Co_x_Fe_3-x_O_4_ with X = 0.05 (1), 0.1 (2), 0.15 (3), 0.2 (4), 0.25 (5), and 0.3 (6). b) Plot of the hysteresis curve area for every sample before and after pre-magnetization, displaying an increase in the hysteresis area for every sample except Co_0.05_Fe_2.95_O_4_ that corresponds to an enhanced induction heating performance.


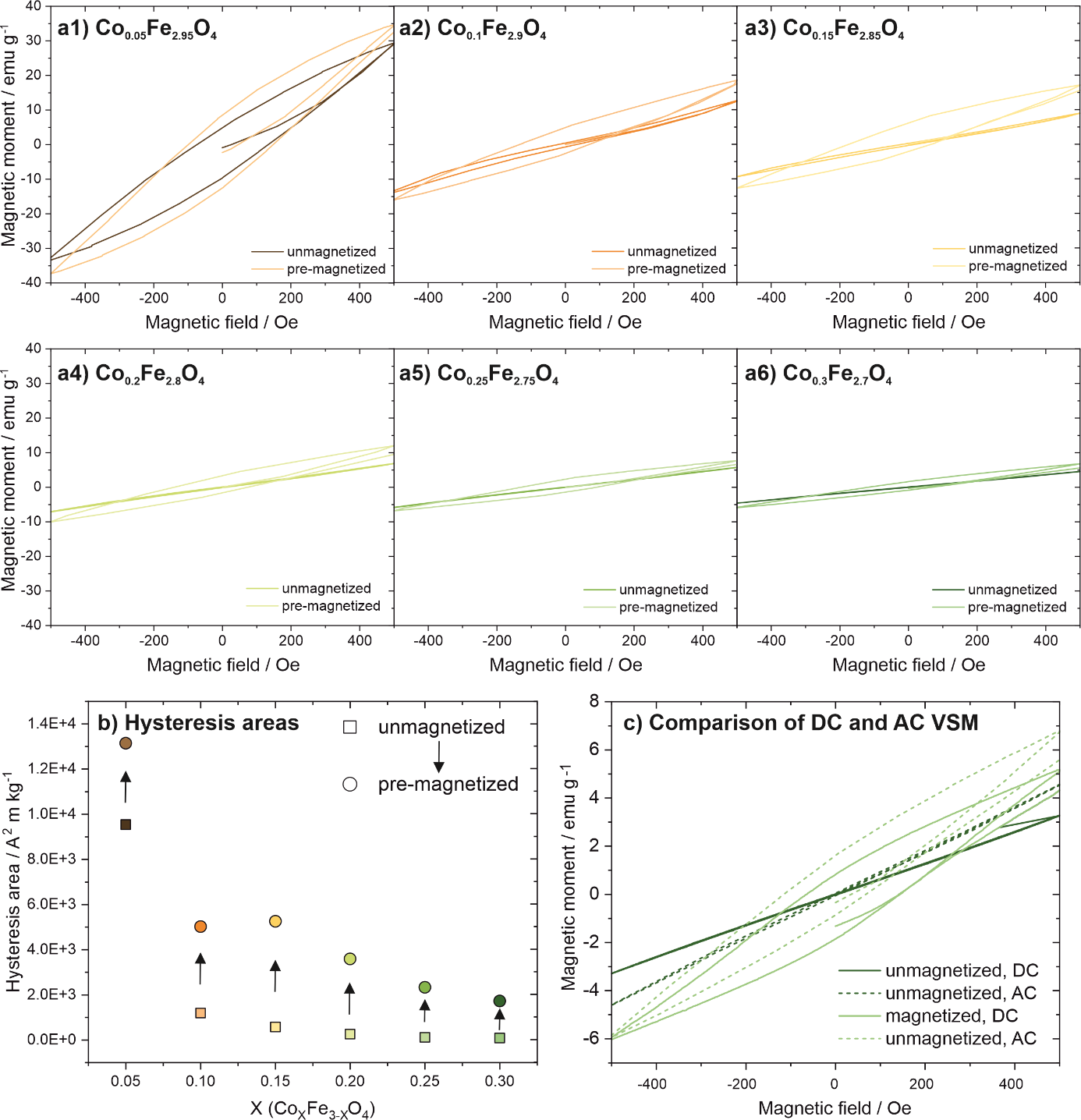


Figure S7. a) Direct comparison of dynamic (1000 Hz) VSM curves of Co-doped FIONs before and after pre-magnetization, recorded between -500 and 500 Oe for NPs of the type Co_x_Fe_3-x_O_4_ with X = 0.05 (1), 0.1 (2), 0.15 (3), 0.2 (4), 0.25 (5), and 0.3 (6). b) Plot of the hysteresis curve area for every sample before and after pre-magnetization, displaying an increase in the hysteresis area for every sample that corresponds to an enhanced induction heating performance. c) Comparison of static and dynamic VSM curves of Co_0.05_Fe_2.95_O_4_ and Co_03_Fe_2.7_O_4_  NPs before and after pre-magnetization recorded between -500 and 500 Oe.

Table S4. Time per temperature rise and energy input given for every Co-doped FION species in the pristine and pre-magnetized state. Data is taken from induction heating curves recorded at a field amplitude of 500 Oe and a frequency of 1419 kHz.

|  | **Pre-magnetized** | | **As prepared** | |
| --- | --- | --- | --- | --- |
|  | **Time / s** | **Energy input / Oe s^-1^ K^-1^** | **Time / s** | **Energy input / Oe s^-1^ K^-1^** |
| **Co_0.05_Fe_2.95_O_4_** | 0.00106 | 0.53 | 0.0020 | 1.0 |
| **Co_0.1_Fe_2.9_O_4_** | 0.00163 | 0.82 | 0.0084 | 42.0 |
| **Co_0.15_Fe_2.85_O_4_** | 0.00175 | 0.88 | 0.0071 | 35.5 |
| **Co_0.2_Fe_2.8_O_4_** | 0.00425 | 2.13 | 0.0175 | 87.5 |
| **Co_0.25_Fe_2.75_O_4_** | 0.00400 | 2.00 | 0.8240 | 412.0 |
| **Co_0.3_Fe_2.7_O_4_** | 0.00233 | 1.17 | 1.0400 | 520.0 |
| **Average** | **0.00250** | **1.26** | **0.3660** | **183.0** |

Table S5. Coercivity and saturation magnetization values obtained from VSM measurements between -500 and 500 Oe for Co_x_Fe_3-x_O_4_ with X = 0.05 to 0.3. Note that, while when recording VSM curves for these samples in a larger static field range, the coercivity is increasing with increasing Co doping, the trend for the minor curves recorded here is opposing. The reason for that is that the hysteresis curves cannot be fully recorded in the given small field, hence the smaller coercivity value here indicates that the respective NP species is not fully magnetizable.

|  | **Hysteresis curve area**  **of AC field** | | **Hysteresis curve area**  **DC field** | | **AC vs DC Hysteresis Area** | |
| --- | --- | --- | --- | --- | --- | --- |
| **X** | **Before magn.** | **After magn.** | **Before magn.** | **After**  **magn.** | **Before**  **magn.** | **After**  **magn.** |
| **0.05** | 9536 | 13147 | 11471 | 8832 | -1935 | 4315 |
| **0.1** | 1195 | 5024 | 1057 | 6797 | 138 | -1773 |
| **0.15** | 578 | 5253 | 552 | 4594 | 26 | 659 |
| **0.2** | 258 | 3593 | 226 | 1728 | 32 | 1865 |
| **0.25** | 114 | 2329 | 109 | 1923 | 5 | 406 |
| **0.3** | 82 | 1724 | 36 | 1667 | 46 | 57 |

**Discussion of the comparison of hysteresis curve area before and after pre-magnetization deduced from measurements with DC and AC fields**

AC hysteresis loop measurements at 1000 Hz reveal a significant increase in hysteresis area after pre-magnetization across nearly all Co doping levels. This is consistent with magnetic pre-conditioning aligning blocked or disordered domains, thereby enhancing energy dissipation under alternating fields. Although 1000 Hz is well below the induction heating frequency (~1.4 MHz), the relative trend provides valuable qualitative insight.

Except for the outlier at X = 0.1, the increase in AC hysteresis area follows a coherent pattern: higher Co content samples initially show low hysteresis losses that rise markedly after pre-magnetization. This supports the interpretation that pre-alignment helps overcome magnetic frustration or anisotropy barriers, especially in highly doped samples.

Minor deviations observed at X = 0.1 (AC) and X = 0.2 (DC) do not undermine the overall consistency between static and dynamic measurements. Together, these results confirm that pre-magnetization enhances dynamic losses, particularly in samples with initially negligible heating efficiency.

It is important to note that, as described by Jiles,^[7]^ frequency-dependent inductive effects tend to vanish for nanoscale particles due to their small cross-sectional area, resulting in largely stable hysteresis loop behavior up to high frequencies. This supports our view that even AC measurements at lower frequency provide meaningful insight into dynamic heating trends.

Thus, despite the frequency limitations, the AC hysteresis loop area at 1000 Hz remains a meaningful proxy for heating performance trends in our system.


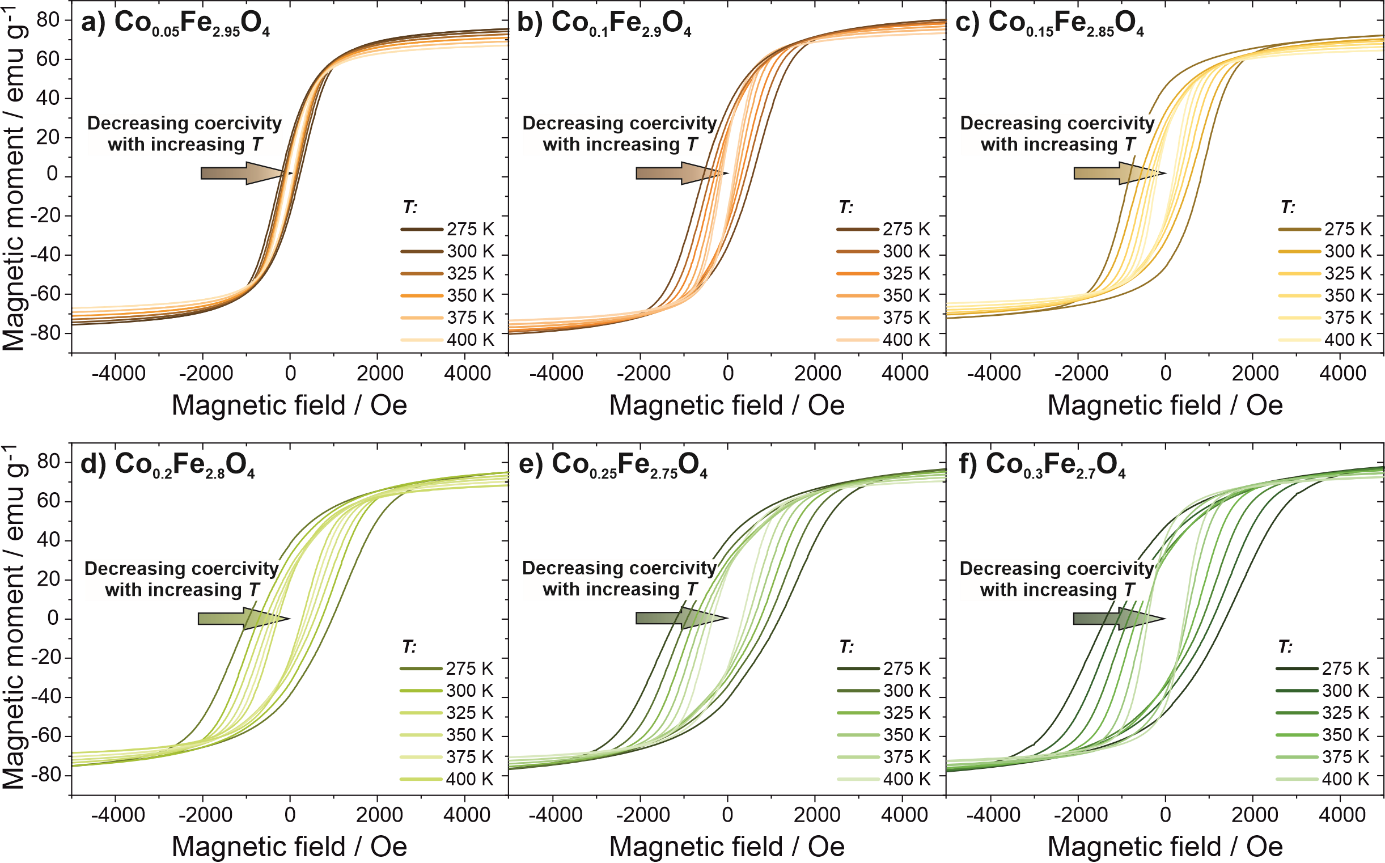


Figure S8. VSM curves recorded at temperatures between 275 and 400 K for NPs of the type Co_x_Fe_3-x_O_4_ with X = 0.05 (a), 0.1 (b), 0.15 (c), 0.2 (d), 0.25 (e), 0.3 (f). For each NP species, the coercivity is decreased with increasing temperature. This behavior is transferrable to the diminished coercivity during induction heating as the NPs become increasingly hot. For clarity, initial curves are not depicted.


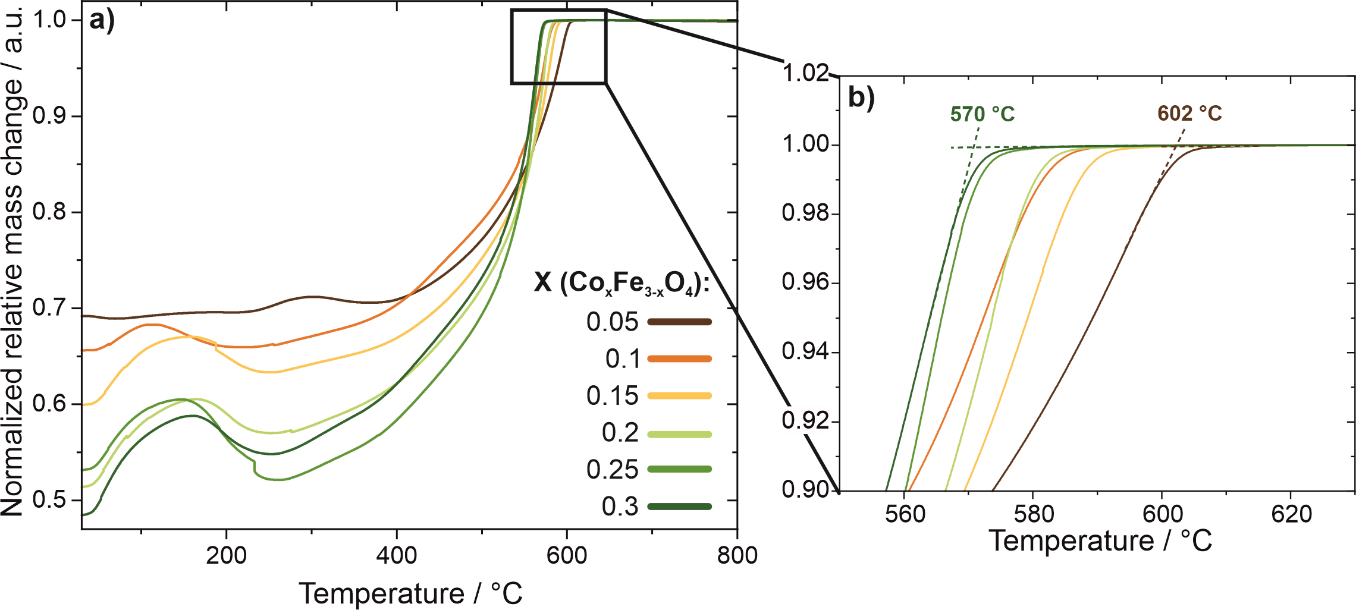


Figure S9. a) Curie temperatures measured for Co-doped FIONs as per TGA with a magnetic attachment. b) Magnification displays the Curie temperature of all samples roughly comparable between 570 and 600 °C. TGA measurements have been performed on pre-magnetized NPs so as to ensure that the barrier provided by high coercivities does not hinder the Curie temperature determination.

Table S6. Coercivity values for Co-doped FIONs of the type Co_x_Fe_3-x_O_4_, obtained from static (DC) VSM measurements between -30 and 30 kOe at different temperatures between 275 and 400 K. The coercivity is decreasing for increasing temperature in each case.

| **X =** | **275 K** | **300 K** | **325 K** | **350 K** | **375 K** | **400 K** |
| --- | --- | --- | --- | --- | --- | --- |
| **0.05** | 215 | 150 | 125 | 100 | 85 | 65 |
| **0.1** | 560 | 405 | 304 | 224 | 170 | 130 |
| **0.15** | 815 | 575 | 430 | 325 | 255 | 188 |
| **0.2** | 960 | 715 | 560 | 440 | 355 | 275 |
| **0.25** | 1180 | 935 | 745 | 590 | 460 | 330 |
| **0.3** | 1370 | 1080 | 845 | 650 | 480 | 420 |

Table S7. Values of the anisotropy constant *K* for Co-doped FIONs of the type Co_x_Fe_3-x_O_4_, obtained from static (DC) VSM measurements between -30 and 30 kOe at different temperatures between 275 and 400 K. The anisotropy constant is decreasing for increasing temperature in each case.

| **X =** | **275 K** | **300 K** | **325 K** | **350 K** | **375 K** | **400 K** |
| --- | --- | --- | --- | --- | --- | --- |
| 0.05 | 17 245 | 11 875 | 9 635 | 7 604 | 6 286 | 4 604 |
| 0.1 | 47 250 | 33 750 | 25 017 | 18 200 | 13 458 | 10 021 |
| 0.15 | 61 974 | 42 526 | 31 354 | 23 021 | 17 797 | 12 729 |
| 0.2 | 76 000 | 56 604 | 43 167 | 33 458 | 26 255 | 19 766 |
| 0.25 | 97 104 | 76 943 | 59 755 | 46 094 | 35 458 | 24 750 |
| 0.3 | 112 740 | 87 750 | 68 656 | 52 135 | 37 500 | 31 938 |

**
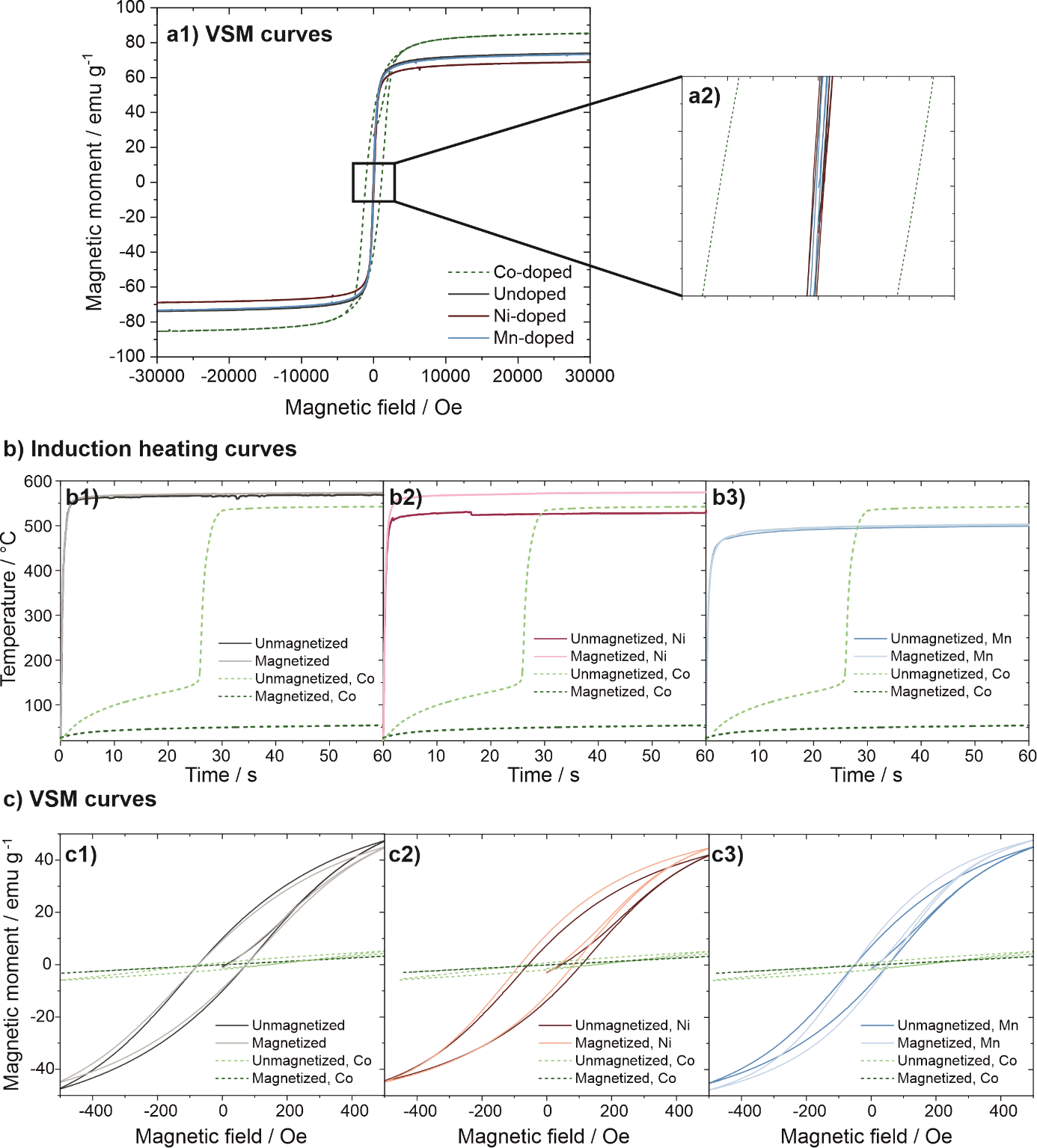
**

Figure S10. Comparison of Co-doped FIONs with other undoped (1) as well as Ni- (2) and Mn-doped (3) species to elucidate whether the programmable switching upon magnetization also works for other NPs. a) Static (DC) VSM curves displaying a significantly larger coercivity for Co-doped species compared to other FIONs. b) Induction heating curves recorded at 500 Oe and 1419 kHz comparing the pristine and pre-magnetized states of all NP species in comparison with Co-doped FIONs. c) Static (DC) VSM curves recorded between -500 and 500 Oe comparing the hysteresis loop in the pre-magnetized and programmed state with those of Co-doped FIONs. The data shows that the desired switching upon pre-magnetization is only possible with Co-doped FIONs due to their significantly enhanced coercivity, introducing a large anisotropy.

**
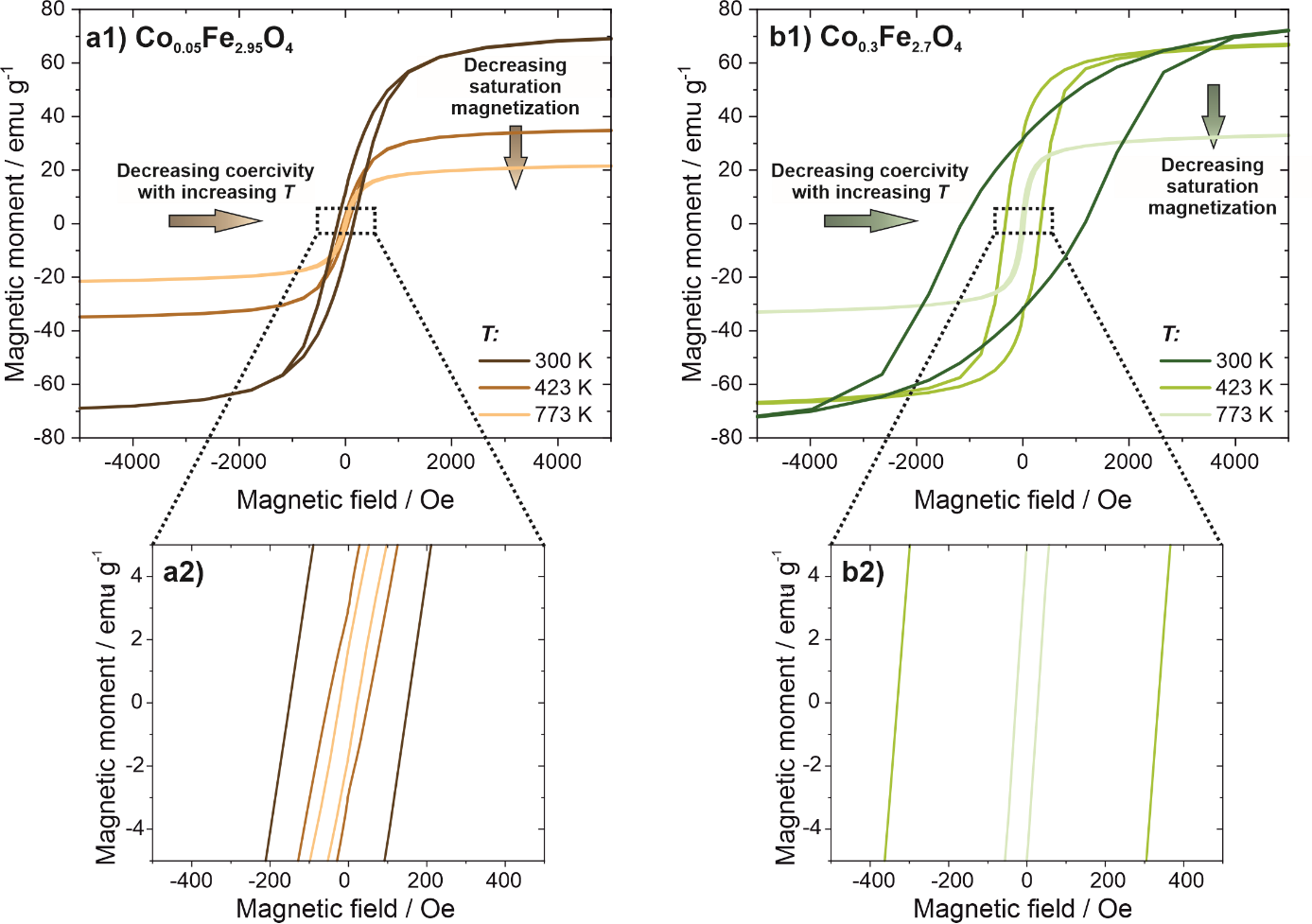
**

Figure S11. High-temperature static (DC) VSM curves recorded at 300, 423 and 773 K for Co_0.05_Fe_2.95_O_4_ (a) and Co_0.3_Fe_2.7_O_4_ NPs (b), respectively. Curves for both species reveal that the saturation magnetization and coercivity both decrease with increasing temperature, however, even at 773 K, there is still a remaining measurable coercivity. For clarity, initial curves are not depicted.

**Discussion of the form of minor hysteresis curves obtained for different pre-magnetization values**

It becomes obvious that with higher pre-magnetization values and higher Co dopings in general, the hysteresis loops recorded between -500 and 500 Oe for Co-doped FIONs become more asymmetric in terms of fanning out, *i.e.* opening up in the direction of positive applied magnetic fields. This phenomenon is due to the applied pre-magnetization being performed in the VSM, meaning that the initial magnetization is recorded along the same direction in which the sample was previously magnetized. Therefore, this results in a higher magnetic saturation compared to when the samples are magnetized in the opposite direction (-500 Oe), causing the magnetic domains to align accordingly. However, when the previously applied pre-magnetization field was higher, the material might not reach a fully saturated state when magnetized in the opposite direction (*e.g.* +500 Oe). As a result, some domains that were priorly aligned do not fully transition back to the saturated state. This misalignment causes an offset between the initial magnetic state and the state after completing one loop, leading to open hysteresis curves.

**Effect of heating Co-doped FIONs to their Curie temperature**

Interestingly, once the Co-doped FIONs are inductively heated up to their Curie temperature, their stimulation towards induction heating is facilitated afterwards. This is in accordance with corresponding hysteresis loops, displaying a larger hysteresis area in the inductively heated compared to the pristine state (1115 vs. 36 A^2^ m kg^‑1^, see Supporting Information, **Table S5** and **Figure S8**). The hysteresis area after induction heating is comparable to that of NPs warmed to their Curie temperature in an oven (1145 A^2^ m kg^‑1^ after 5 min at 700 °C), indicating that the Curie point was met during oven as well as induction heating. The reduction of the hysteresis area for Curie-heated samples in comparison to pre-magnetized, unheated NPs (here: 1667 A^2^ m kg^‑1^) is presumably due to oxidation processes of magnetite.^[8]^
A potential reason for the altered induction heating behavior after annealing the sample to its Curie temperature could be that the pristine state of the NPs is not randomly magnetized, as limited magnetic flux closure could occur during the growth of the particles, which is however not the energetically lowest state. Flux closure is defined as a condition with a confined magnetostatic field that exhibits a zero-moment vortex state,^[9]^ caused by high dipolar interactions and spontaneous assembly under zero field to reduce the magnetostatic energy.^[10]^ This state is lost once the NPs are thermally annealed, causing the sample to be fully magnetically rearranged once it was heated to its Curie temperature, in the process “unblocking” a more stable and energetically more favorable domain state.^[11]^ This is further confirmed by considering the hysteresis area of unmagnetized samples heat-treated to their Curie temperature in an oven, which is enlarged compared to that of untreated pristine samples (522 vs. 36 A^2^ m kg^‑1^, see **Table S5** and **Figure S8**).
It is notable that the induction heating behavior of the samples after they have been heated to their Curie temperature in an oven remains the same, even when they are subsequently magnetized (see Supporting Information, **Figure S8c**). This further indicates that the magnetic switching of the induction heating behavior of Co-doped FIONs can be selectively turned off by annealing them. The reason for that presumably lies in the partial oxidation of the Co-doped magnetite samples to Co-doped maghemite at higher temperatures,^[8]^ which is pointed out by the lower activation temperature for induction heating after annealing. Here, induction heating is enabled after heating to temperatures of 50 to 60 °C, while in the pristine state, it is only initiated after reaching temperature levels of approximately 140 °C. As it was discussed earlier, this is the point at which the temperature-dependent anisotropy barrier is overcome. However, this value is lower with Co-doped maghemite compared to magnetite,^[3,12]^ therefore enabling an earlier excitation of the induction heating process. Another possible reason for the differing induction heating behavior after oven treatment could be that defects in the crystal lattice are reduced by annealing, hence reducing anisotropy and resulting energy barriers. This would enable a faster activation of enhanced induction heating rates as anisotropy barriers are easier overcome.

Table S8. Hysteresis loop areas for different states of Co_0.3_Fe_2.7_O_4_ NPs obtained from static (DC) VSM curves, displaying an enhanced hysteresis area after pre-magnetization that is slightly reduced when they are heated to their Curie temperature, albeit comparable between oven and induction heating treatment. Additionally, the hysteresis area is also enlarged when compared untreated and Curie temperature-treated unmagnetized samples. Values given in A^2^ m kg^‑1^.

|  | **Unmagnetized** | **Magnetized** | **Inductively heated** |
| --- | --- | --- | --- |
| **Untreated** | 36 | 1667 | 1115 |
| **Oven-treated** | 522 | 1145 |  |


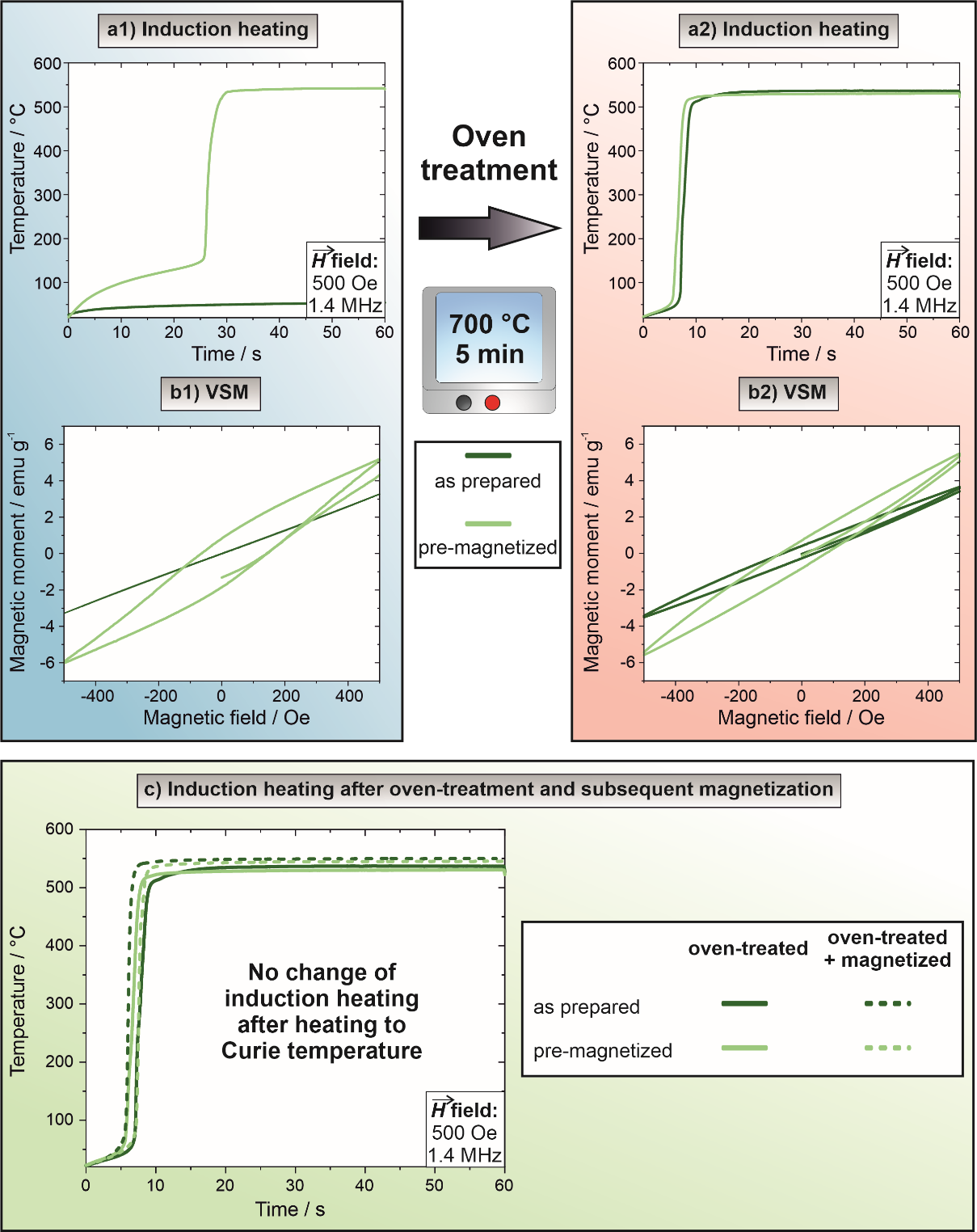


Figure S12. Influence of oven heating to the Curie temperature of Co_0.3_Fe_2.7_O_4_ NPs on their magnetic characteristics. a) Induction heating performance before (1) and after (2) treatment in an oven at 700 °C for 5 min, revealing an enhanced heat production after annealing that is unchanged between the as prepared and magnetized state. b) Static (DC) VSM data before (1) and after (2) oven treatment, indicating an enlarged hysteresis loop for the unmagnetized and a slightly reduced loop for the magnetized state. c) Induction heating performance of oven-treated samples that were magnetized after heating to their Curie temperature. This data indicates that once the sample was heated to its Curie point, its induction heating behavior is not influenced by magnetization with an external magnet anymore. For all induction heating curves, a field amplitude of 500 Oe and an AMF frequency of 1419 kHz were employed.


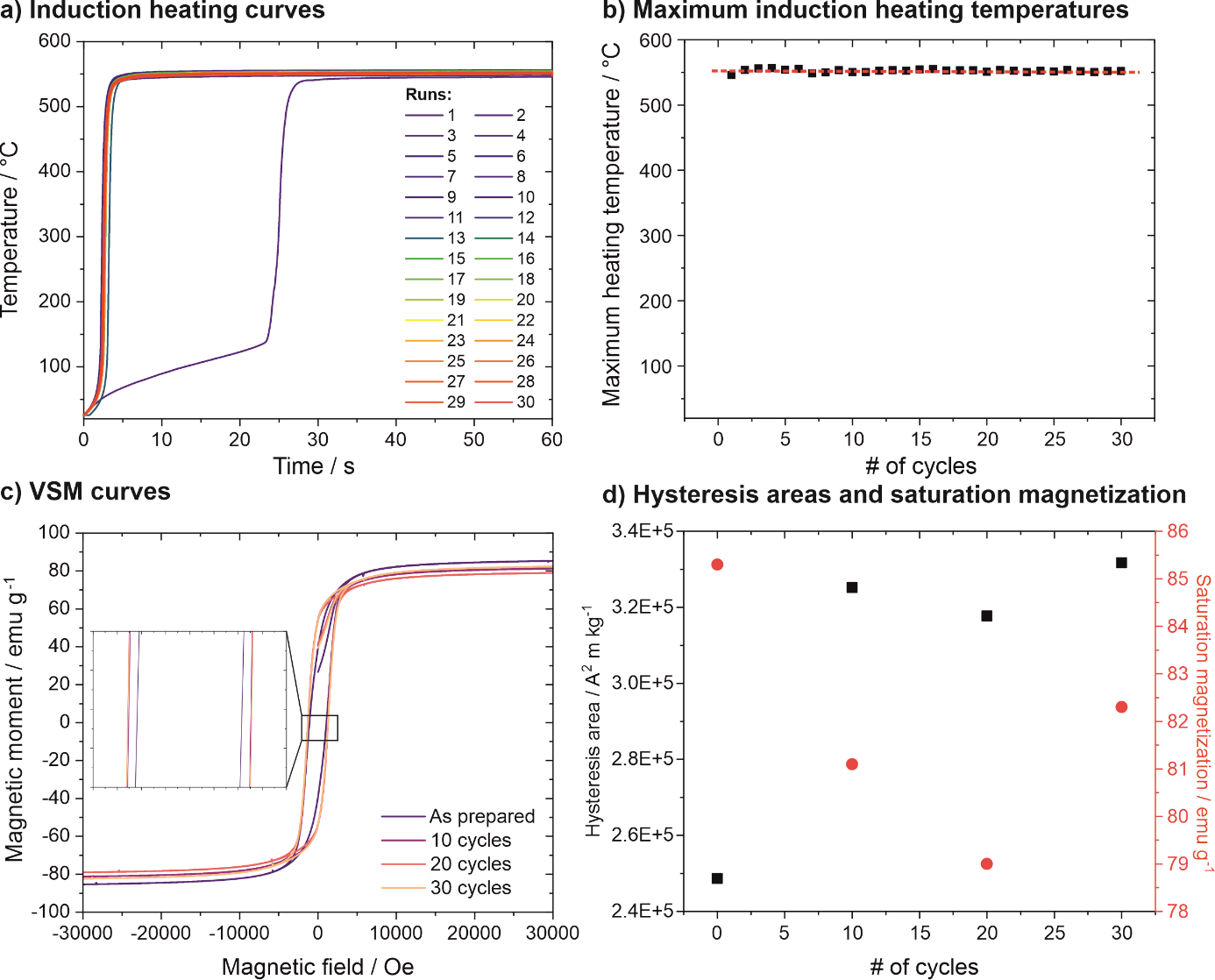


Figure S13. Durability of Co_0.3_Fe_2.7_O_4_ nanoparticles over thirty induction heating cycles. a) Induction heating curves obtained at an AMF amplitude of 500 Oe and a frequency of 1419 kHz. b) Maximum induction heating temperatures obtained from the corresponding induction heating data, showing that the maximum temperature is maintained over all runs. c) Static (DC) VSM curves measured in the pristine state and after every 30 cycles, revealing only a slight decrease in the coercivity. d) Hysteresis area and saturation magnetization changes over thirty consecutive induction heating runs. While the hysteresis area is increased with an increasing number of cycles, the saturation magnetization is diminished until 20 runs, before it is increased again. The maintained maximum heating temperature indicates that activation towards induction heating remains permanent. The acquired datapoints up until 30 cycles do not show any change in durability over repeated heating. While minor changes in the hysteresis loops indicate the nanoparticles undergoing slight changes to their magnetic nature, the effect remains small enough to not interfere with the aimed induction heating behavior. Therefore, we presume that the magnetic alignment is not lost over multiple heating cycles, but rather enhanced by thermal annealing, which manifests in the fixed initial activation for induction heating.

**
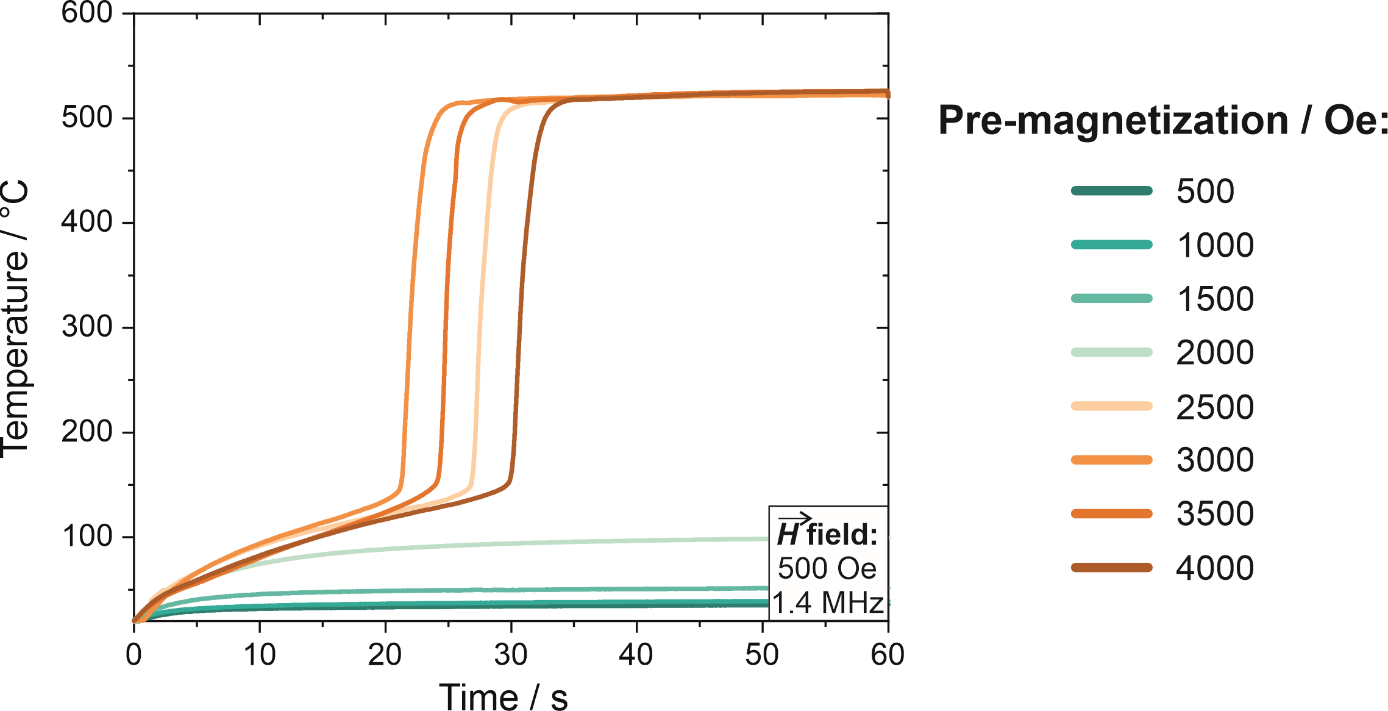
**

Figure S14. Induction heating curves of Co_0.3_Fe_2.7_O_4_ NPs with different pre-magnetization values between 500 and 4000 Oe. Curves were obtained at an AMF amplitude of 500 Oe and a frequency of 1419 kHz. The data shows that respective Co-doped FIONs are stimulated for enhanced induction heating at a minimum pre-magnetization value of 2500 Oe, in accordance with VSM data.

**
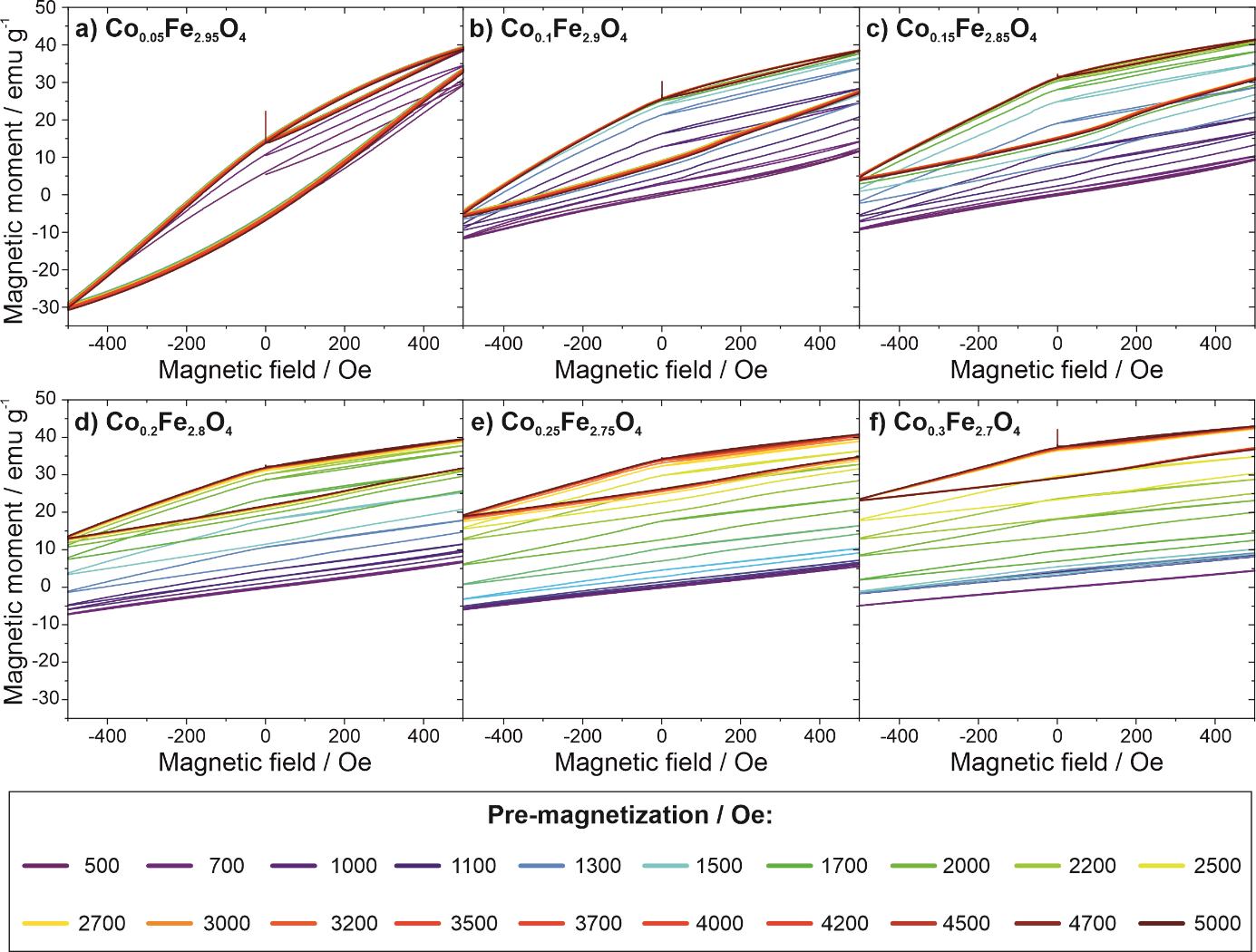
**

Figure S15. Static (DC) VSM curves recorded between -500 and 500 Oe for Co-doped FIONs of the type Co_x_Fe_3-x_O_4_ with X = 0.05 (a), 0.1 (b), 0.15 (c), 0.2 (d), 0.25 (e), and 0.3 (f) after different pre-magnetization values between 500 and 5000 Oe. With lower Co dopings, lower pre-magnetization strength is needed to achieve hysteresis loops that remain unchanged with higher magnetization values, while for all samples, approximately the same magnetic moment of 40 to 45 emu g^‑1^ is reached as a plateau. To achieve the plot of the highest magnetic moment against pre-magnetization, the highest saturation magnetization value for every respective pre-magnetization in every Co-doped FION sample was determined and plotted accordingly.


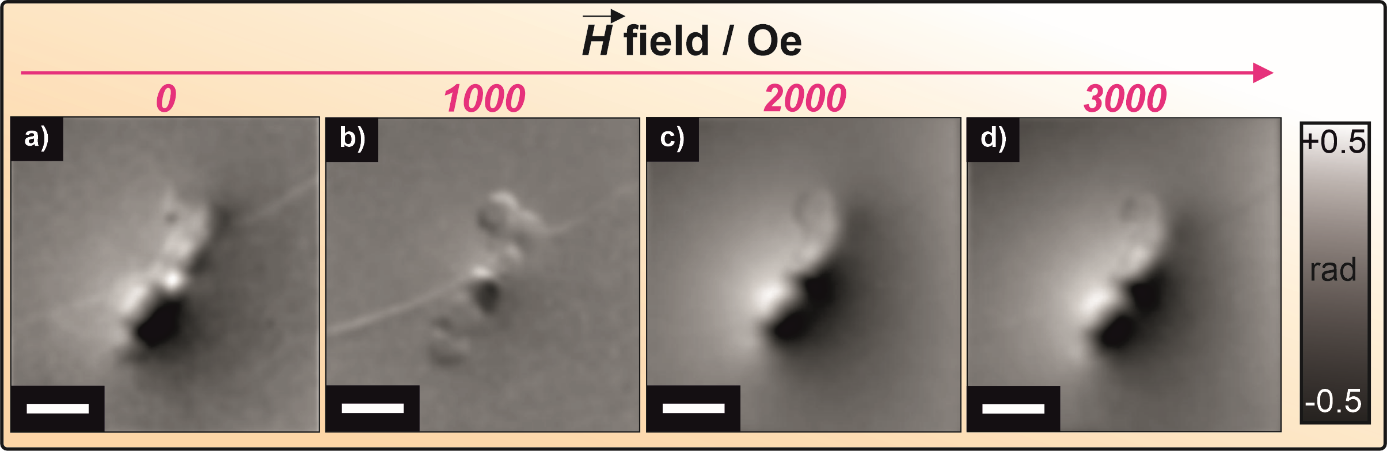


Figure S16. Magnetic phase images of Co_0.3_Fe_2.7_O_4_ NPs extracted from off-axis electron holography recorded at remanent state (zero field) after applying a magnetic field of 0 (a), 1000 (b), 2000 (c), and 3000 Oe (d), respectively. The magnetic field was applied along the NP chain’s long axis. Scale bars correspond to 100 nm.


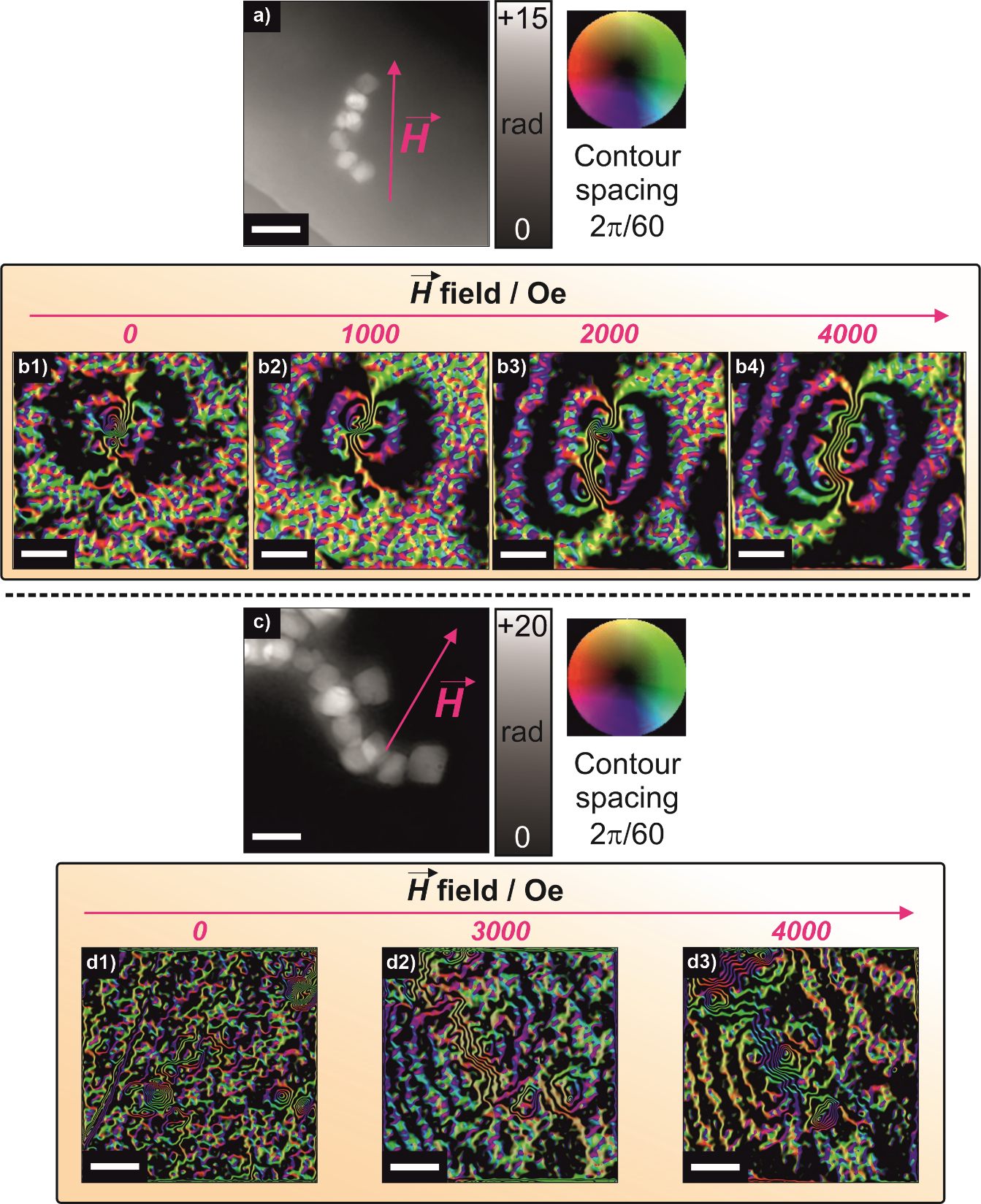


Figure S17. Electrostatic phase shift (a, c) and projected magnetic field distribution (b, d) of Co_0.05_Fe_2.95_O_4_ NPs recorded at remanent state after retracting the external magnetic field measured by off-axis electron holography. The applied magnetic field is parallel (b) and perpendicular (d) to the NP chain axis, respectively. The magnetic moments of Co-doped FIONs are intrinsically oriented non-uniformly and rearrange with exposure to high magnetic fields until their saturated state is reached. Notably, a higher saturated magnetic field is needed for NPs that are aligned perpendicular to the external magnetic field, as shown in d compared to b. Scale bars correspond to 100 nm.

**Discussing of literature regarding programmable enhanced induction heating behavior**

An enhanced induction heating performance of magnetic NPs after pre-alignment has also been reported elsewhere in literature.^[13]^ Here, it is stated that the heat generation of NPs depends on the angle between their easy axis and the applied AMF, while it is maximized when both are aligned. However, these introduced NP systems lack programmable induction heating towards higher temperature thresholds as well as the tunability in terms of the applied AMF that is given here by employing different Co dopings in FIONs. Additionally, it has been published for single domain cubic iron oxide NPs with a high intrinsic anisotropy that dipolar interactions are improved upon self-assembly into chain-like structures. In turn, induction heating properties are enhanced, as the NP assemblies act as a collective, similar to the system presented herein.^[14]^


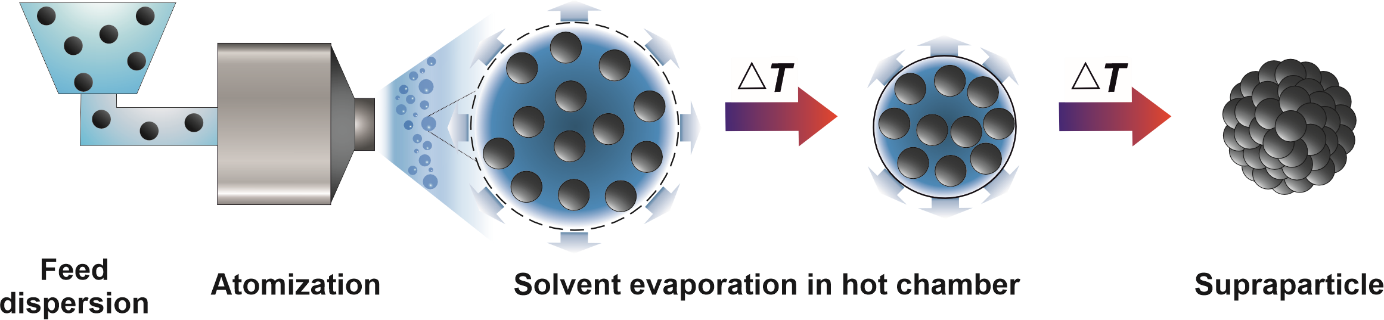


Figure S18. Spray-drying procedure. A NP feed dispersion is fed into a nozzle, where the dispersion is atomized into droplets that are transported into a hot chamber by a gas stream. There, the solvent evaporates, forcing the contents of the droplet, *i.e.* NP building blocks, into higher-hierarchical assemblies, so-called supraparticles (SPs). The resulting SPs are then collected as a fine powder.


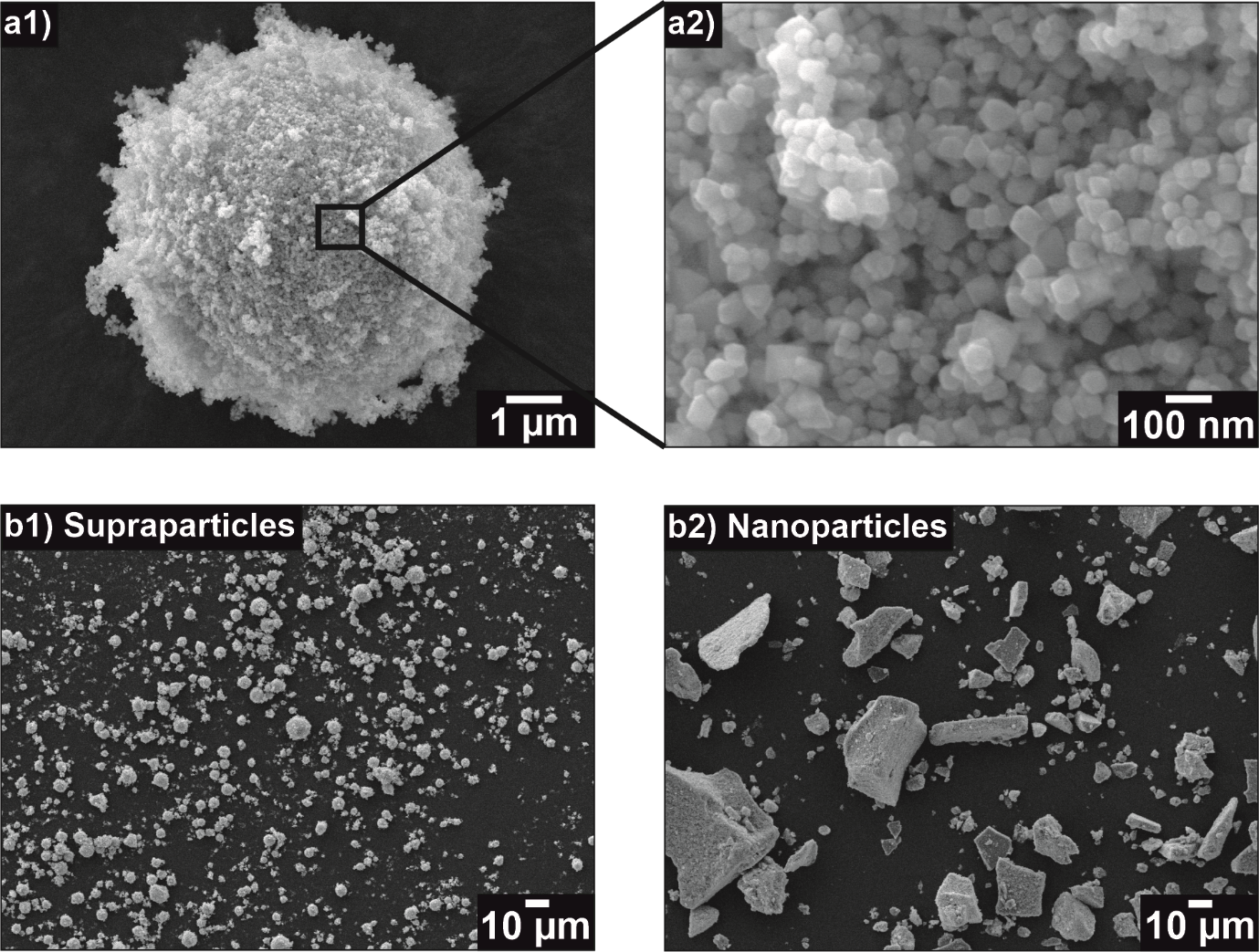


Figure S19. a) SEM images of spray-dried SPs consisting of Co_0.05_Fe_2.95_O_4_ NP building blocks. b) Comparison of SEM images of spray-dried SPs (1) and agglomerated NPs (2) with the same magnification. These images reveal a more defined structure of SPs in comparison with agglomerated NPs in terms of size distribution and uniformity, which justifies the incorporation of SPs into the envisaged composites.


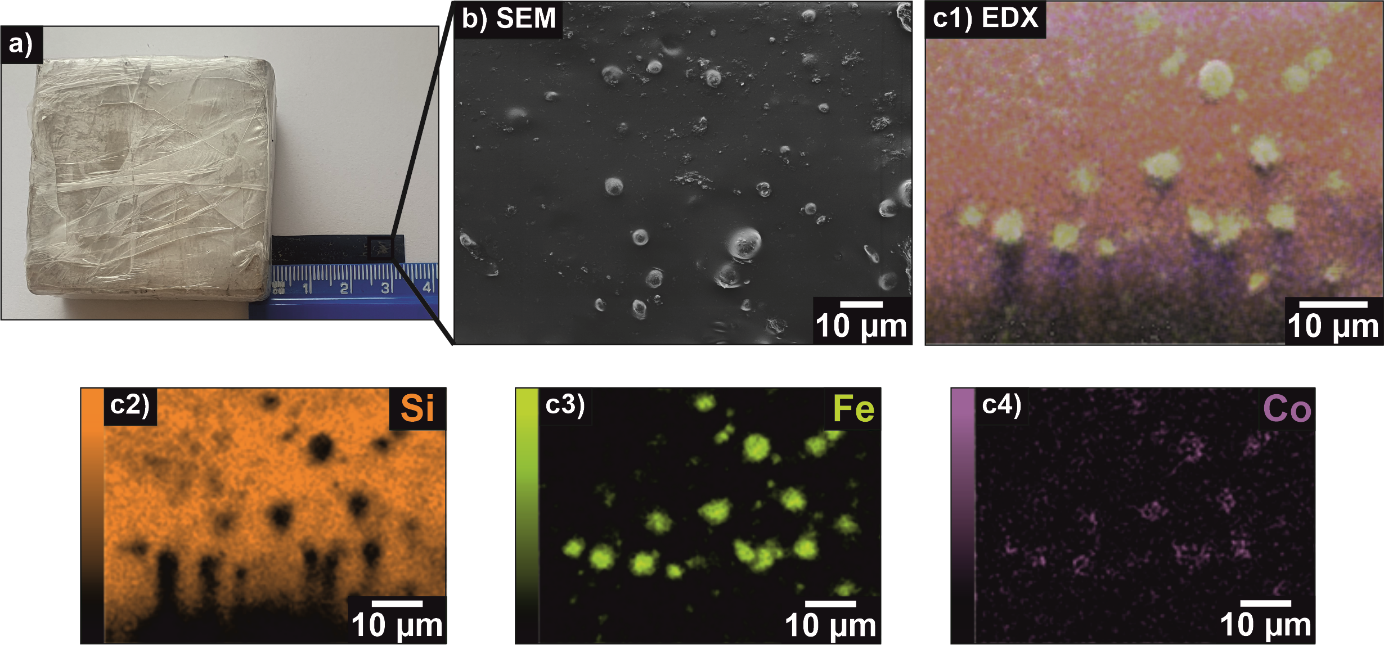


Figure S20. Composite of 30 wt% Co_0.05_Fe_2.95_O_4_ SPs in a PDMS matrix that was locally magnetized with a static magnet (a). SEM (b) reveals a uniform integration of the SPs into the matrix, which is further confirmed by EDX (c1) showing Si (c2) in the PDMS environment and Fe (c3) and Co (c4) in the SPs, correspondingly.


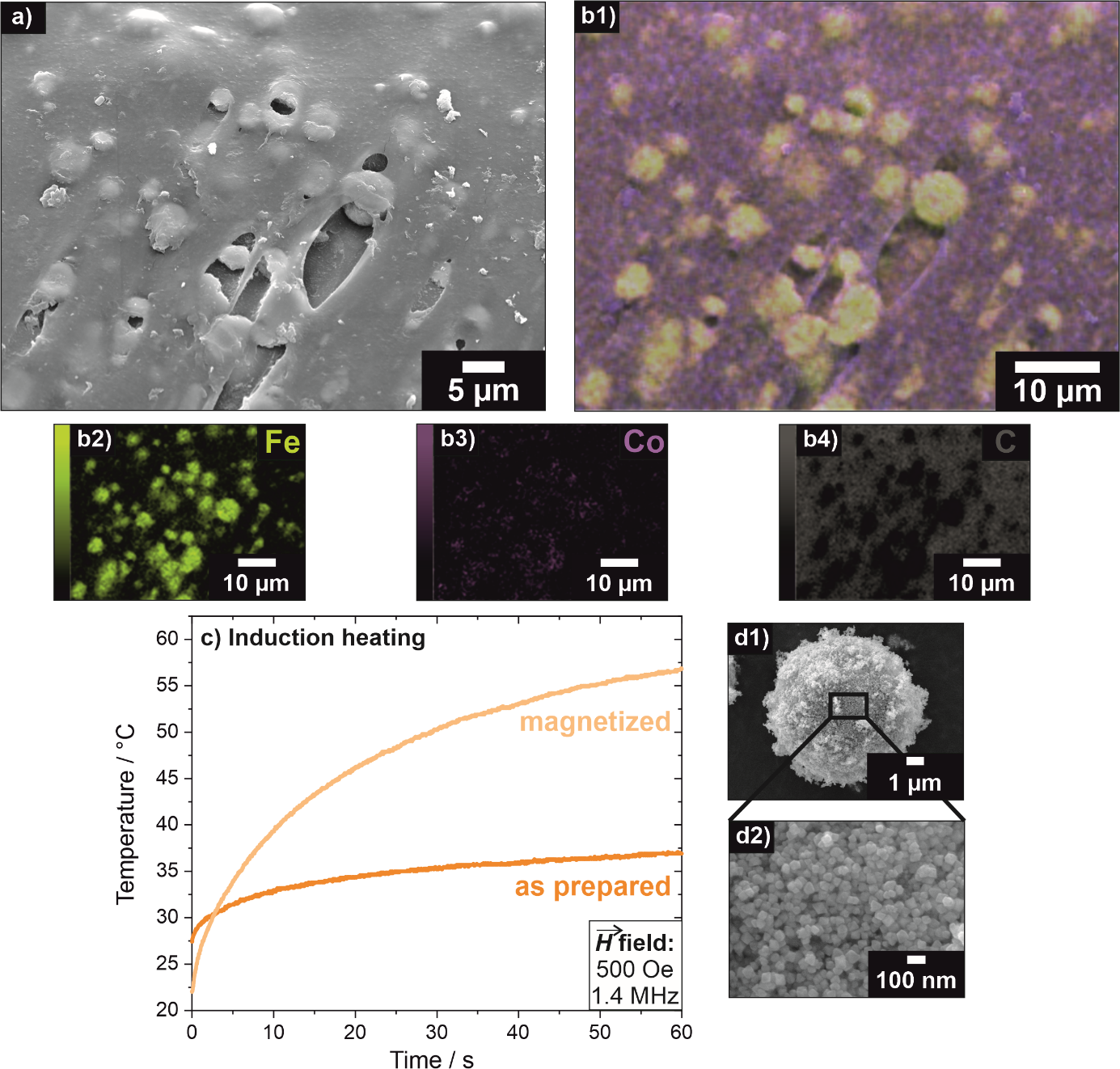


Figure S21. Composite of 20 wt% Co_0.1_Fe_2.9_O_4_ SPs in a hot glue matrix. SEM (a) reveals uniform integration of the SPs into the matrix, which is further confirmed by EDX (b1) where Fe (b2) and Co (b3) are found in the SPs, while C (b4) is only found in the surrounding environment. c) Induction heating curves recorded at a field amplitude of 500 Oe and a frequency of 1419 kHz show a heat production of 55 °C of the magnetized spot, while the pristine, unmagnetized spot only inductively heats to 35 °C. d) SEM images of the incorporated SPs.


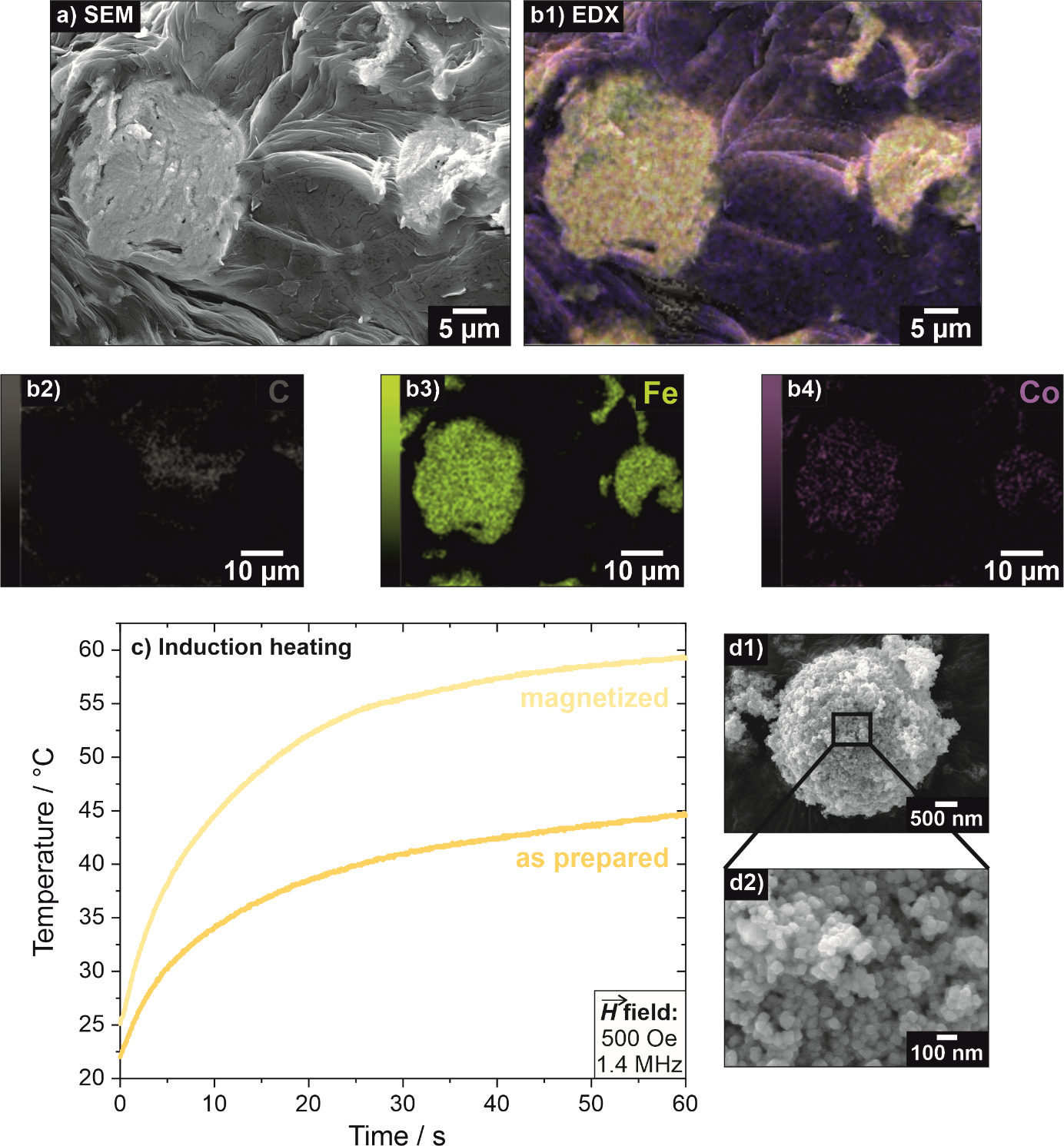


Figure S22. Composite of 20 wt% Co_0.15_Fe_2.85_O_4_ SPs in a PEG matrix. SEM (a) reveals integration of the SPs into the matrix, which is further confirmed by EDX (b1) where C (b2) is found in the surrounding environment, while Fe (b3) and Co (b4) are found in the SPs. c) Induction heating curves recorded at a field amplitude of 500 Oe and a frequency of 1419 kHz show a heat production of 60 °C of a magnetized crucible, while the unmagnetized crucible only inductively heats to 45 °C. d) SEM images of the incorporated SPs.

Discussion of the correlation between temperatures profiles in induction heating and the thermal conductivity of the host matrix

Co_0.05_Fe_2.95_O_4_ particles were integrated into PDMS (thermal conductivity: ~0.2 W m^-1^ K^-1^),^[15]^ Co_0.1_Fe_2.9_O_4_ into a hot glue based on ethylene vinyl acetate copolymer (thermal conductivity: ~0.28 – 0.32 W m^-1^ K^-1^),^[16]^ and Co_0.15_Fe_2.85_O_4_ particles into PEG (thermal conductivity: ~0.33 W m^-1^ K^-1^).^[17]^ It is notable that the Co doping of the employed particles is chosen to increase with increasing thermal conductivity of the selected matrix. By directly comparing the induction heating curves of all particle species in the different matrices with one another, the differing thermal conductivities of the polymer materials are imminent already in the unmagnetized state (**Figure S20a**). While temperature profiles of Co_0.05_Fe_2.95_O_4_ in PDMS and Co_0.1_Fe_2.9_O_4_ in hot glue are similar, particles incorporated into PEG display a significantly larger overall temperature difference despite their higher Co doping. In the pre-magnetized state, the lowest Co-doped particles display the highest overall temperature and the highest heating rate (**Figure S20b**). However, pre-magnetized Co_0.15_Fe_2.85_O_4_ in PEG show a higher reached temperature during induction heating compared to the Co_0.1_Fe_2.9_O_4_ particles integrated into hot glue. These results indicate that the thermal conductivity of the matrix material has an effect on the temperature profile of integrated Co-doped FIONs during induction heating. In the pristine state, a higher thermal conductivity of the environment leads to enhanced heating rates. In the pre-magnetized state, heating rates of lower doped particles are high independent of the thermal conductivity of the surroundings, however, with higher dopings, induction heating performance can be improved by choosing a material with a high thermal conductivity.

**
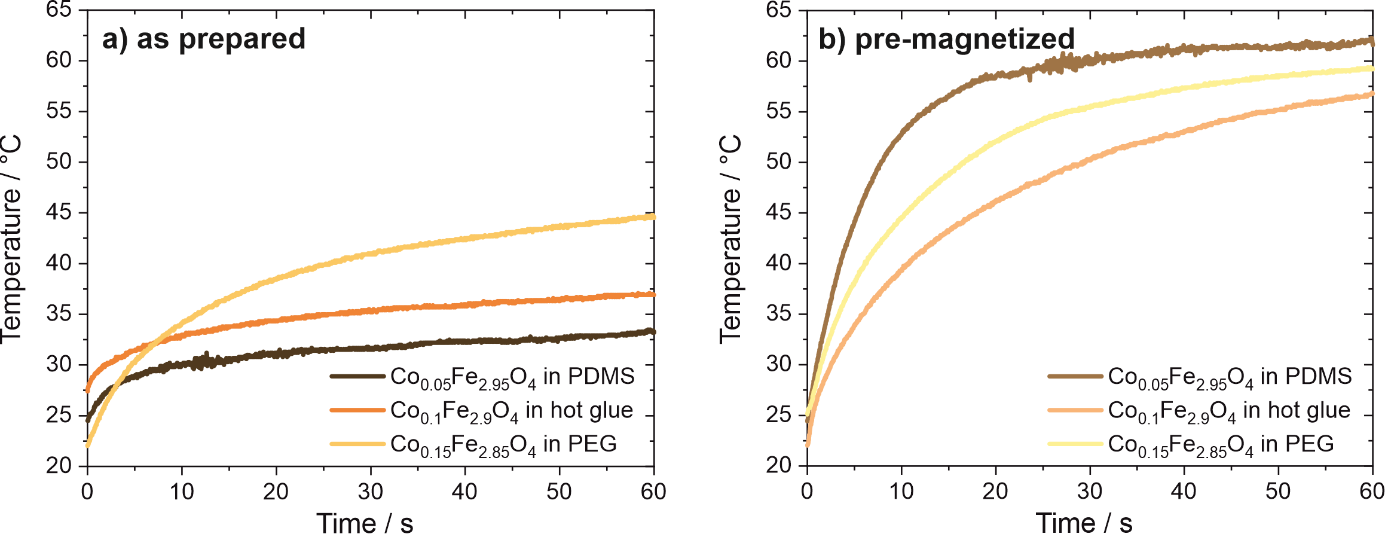
**

Figure S23. Induction heating curves obtained at an amplitude of 500 Oe and a frequency of 1419 kHz for different Co-doped FION species incorporated into PDMS, hot glue, and PEG matrices, shown in the pristine (a) and pre-magnetized (b) state. Differing thermal conductivities of the matrix materials have an influence on the induction heating profiles, reaching higher heating rates and overall temperature differences with increasing thermal conductivity.

References

[1]  S. Anjum, R. Tufail, K. Rashid, R. Zia, S. Riaz, *J. Magn. Magn. Mater.* **2017**, *432*, 198-207.

[2]  Z. E. Gahrouei, S. Labbaf, A. Kermanpur, *Phys. E: Low-Dimens. Syst. Nanostructures* **2020**, *116*, 113759.

[3]  L. T. H. Phong, D. H. Manh, P. H. Nam, V. D. Lam, B. X. Khuyen, B. S. Tung, T. N. Bach, D. K. Tung, N. X. Phuc, T. V. Hung, T. L. Mai, T.-L. Phan, M. H. Phan, *RSC Adv.* **2021**, *12*, 698-707.

[4]  N. Song, S. Gu, Q. Wu, C. Li, J. Zhou, P. Zhang, W. Wang, M. Yue, *J. Magn. Magn. Mater.* **2018**, *451*, 793-798.

[5]  A. López-Ortega, E. Lottini, C. d. J. Fernández, C. Sangregorio, *Chem. Mater.* **2015**, *27*, 4048-4056.

[6]  A. R. Yasemian, M. Almasi Kashi, A. Ramazani, *Mater. Res. Express* **2020**, *7*, 16113.

[7]  D. C. Jiles, *J. Appl. Phys.* **1994**, *76*, 5849-5855.

[8]  J. R. Correa, D. Canetti, R. Castillo, J. C. Llópiz, J. Dufour, *Mater. Res. Bull.* **2006**, *41*, 703-713.

[9]  M. W. Szyndler, R. M. Corn, *J. Phys. Chem. Lett.* **2012**, *3*, 2320-2325.

[10] a) P. Guardia, A. Labarta, X. Batlle, *J. Phys. Chem. C* **2011**, *115*, 390-396; b) D. Toulemon, Y. Liu, X. Cattoën, C. Leuvrey, S. Bégin-Colin, B. P. Pichon, *Langmuir* **2016**, *32*, 1621-1628.

[11] T. P. Almeida, A. R. Muxworthy, A. Kovács, W. Williams, P. D. Brown, R. E. Dunin-Borkowski, *Sci. Adv.* **2016**, *2*, e1501801.

[12] a) E. H. Sánchez, M. Vasilakaki, S. S. Lee, P. S. Normile, M. S. Andersson, R. Mathieu, A. López-Ortega, B. P. Pichon, D. Peddis, C. Binns, P. Nordblad, K. Trohidou, J. Nogués, J. A. de Toro, *Small* **2022**, *18*, e2106762; b) B. Gross, S. Philipp, E. Josten, J. Leliaert, E. Wetterskog, L. Bergström, M. Poggio, *Phys. Rev. B* **2021**, *103*, 014402.

[13] a) M. M. Beck, C. Lammel, B. Gleich, *J. Magn. Magn. Mater.* **2017**, *427*, 195-199; b) Z. Sha, X. Cheng, A. D. Charles, Y. Zhou, M. S. Islam, A. N. Rider, S. Peng, M. Lim, V. Timchenko, C. H. Wang, *Compos. Struct.* **2023**, *321*, 117304; c) I. Conde-Leborán, D. Serantes, D. Baldomir, *J. Magn. Magn. Mater.* **2015**, *380*, 321-324; d) Y. Yang, X. Liu, Y. Lv, T. S. Herng, X. Xu, W. Xia, T. Zhang, J. Fang, W. Xiao, J. Ding, *Adv. Funct. Mater.* **2015**, *25*, 812-820.

[14] C. Martinez-Boubeta, K. Simeonidis, A. Makridis, M. Angelakeris, O. Iglesias, P. Guardia, A. Cabot, L. Yedra, S. Estradé, F. Peiró, Z. Saghi, P. A. Midgley, I. Conde-Leborán, D. Serantes, D. Baldomir, *Sci. Rep.* **2013**, *3*, 1652.

[15] T. Luo, K. Esfarjani, J. Shiomi, A. Henry, G. Chen, *J. Appl. Phys.* **2011**, *109*, 074321.

[16] Y. Jia, J. Zhang, *Thermochim. Acta* **2022**, *708*, 179141.

[17] Y. Kou, S. Wang, J. Luo, K. Sun, J. Zhang, Z. Tan, Q. Shi, *J. Chem. Thermodyn.* **2019**, *128*, 259-274.
